# LINC00173.v1 promotes angiogenesis and progression of lung squamous cell carcinoma by sponging miR-511-5p to regulate VEGFA expression

**DOI:** 10.1186/s12943-020-01217-2

**Published:** 2020-05-30

**Authors:** Jiarong Chen, Aibin Liu, Zhihui Wang, Bin Wang, Xingxing Chai, Wenjie Lu, Ting Cao, Ronggang Li, Minyan Wu, Zhuming Lu, Wenguang Pang, Lin Xiao, Xiangmeng Chen, Yan Zheng, Qiong Chen, Jincheng Zeng, Jun Li, Xin Zhang, Dong Ren, Yanming Huang

**Affiliations:** 1 Clinical Experimental Center, Jiangmen Key Laboratory of Clinical Biobanks and Translational Research, Jiangmen Central Hospital, Affiliated Jiangmen Hospital of Sun Yat-sen University, Jiangmen, 529030 China; 2 Department of Oncology, Jiangmen Central Hospital, Affiliated Jiangmen Hospital of Sun Yat-sen University, Jiangmen, 529030 China; 3 Department of Geriatrics, Xiangya Hospital, Central South University, Changsha, 410008 China; 4 National Clinical Research Center for Geriatric Disorders, Xiangya Hospital, Central South University, Changsha, 410008 China; 5 Dongguan Key Laboratory of Medical Bioactive Molecular Developmental and Translational Research, Guangdong Provincial Key Laboratory of Medical Molecular Diagnostics, Guangdong Medical University, Dongguan, 523808 China; 6 Collaborative Innovation Center for Antitumor Active Substance Research and Development, Guangdong Medical University, Zhanjiang, 524023 China; 7 Laboratory Animal Center, Guangdong Medical University, Zhanjiang, 524023 China; 8 Department of Pathology, Jiangmen Central Hospital, Affiliated Jiangmen Hospital of Sun Yat-sen University, Jiangmen, 529030 China; 9 Department of Basic Medicine, Guangdong Jiangmen Chinese Medical College, Jiangmen, 529030 China; 10 Department of Thoracic Surgery, Jiangmen Central Hospital, Affiliated Jiangmen Hospital of Sun Yat-sen University, Jiangmen, 529030 China; 11 Department of Radiotherapy Center, Jiangmen Central Hospital, Affiliated Jiangmen Hospital of Sun Yat-sen University, Jiangmen, 529030 China; 12 Department of Radiology, Jiangmen Central Hospital, Affiliated Jiangmen Hospital of Sun Yat-sen University, Jiangmen, 529030 China; 13 Department of Research and Development, Research and Development Center for Molecular Diagnosis Engineering Technology of Human Papillomavirus (HPV) Related Diseases of Guangdong Province, Hybribio Limited, Chaozhou, 521021 China

**Keywords:** LINC00173.v1, miR-511-5p, VEGFA, Angiogenesis, Lung squamous cell carcinoma

## Abstract

**Background:**

Anti-angiogenic therapy represents a promising strategy for non-small-cell lung cancer (NSCLC) but its application in lung squamous cell carcinoma (SQC) is limited due to the high-risk adverse effects. Accumulating evidence indicates that long noncoding RNAs (lncRNAs) mediate in tumor progression by participating in the regulation of VEGF in NSCLC, which might guide the development of new antiangiogenic strategies.

**Methods:**

Differential lncRNA expression in SQC was analyzed in AE-meta and TCGA datasets, and further confirmed in lung cancer tissues and adjacent normal tissues with RT-qPCR and in-situ hybridization. Statistical analysis was performed to evaluate the clinical correlation between LINC00173.v1 expression and survival characteristics. A tube formation assay, chick embryo chorioallantoic membrane assay and animal experiments were conducted to detect the effect of LINC00173.v1 on the proliferation and migration of vascular endothelial cells and tumorigenesis of SQC in vivo. Bioinformatics analysis, RNA immunoprecipitation and luciferase reporter assays were performed to elucidate the downstream target of LINC00173.v1. The therapeutic efficacy of antisense oligonucleotide (ASO) against LINC00173.v1 was further investigated in vivo. Chromatin immunoprecipitation and high throughput data processing and visualization were performed to identify the cause of LINC00173.v1 overexpression in SQC.

**Results:**

LINC00173.v1 was specifically upregulated in SQC tissues, which predicted poorer overall and progression-free survival in SQC patients. Overexpression of LINC00173.v1 promoted, while silencing LINC00173.v1 inhibited the proliferation and migration of vascular endothelial cells and the tumorigenesis of SQC cells in vitro and in vivo. Our results further revealed that LINC00173.v1 promoted the proliferation and migration of vascular endothelial cells and the tumorigenesis of SQC cells by upregulating VEGFA expression by sponging miR-511-5p. Importantly, inhibition of LINC00173.v1 via the ASO strategy reduced the tumor growth of SQC cells, and enhanced the therapeutic sensitivity of SQC cells to cisplatin in vivo. Moreover, our results showed that squamous cell carcinoma-specific factor ΔNp63α contributed to LINC00173.v1 overexpression in SQC.

**Conclusion:**

Our findings clarify the underlying mechanism by which LINC00173.v1 promotes the proliferation and migration of vascular endothelial cells and the tumorigenesis of SQC, demonstrating that LINC00173.v1-targeted drug in combination with cisplatin may serve as a rational regimen against SQC.

## Introduction

Lung cancer is the most commonly diagnosed cancer and the leading cause of cancer-related deaths worldwide. According to Global Cancer Statistics 2018, lung cancer accounts for 11.6% of all cancer cases and 18.4% of all cancer-related deaths [1], and the 5-year survival rate has been reported to be less than 18% [2]. Pathologically, there are two main histological types of lung cancer, including non-small cell lung cancer (NSCLC) and small cell lung cancer (SCLC). NSCLC can be further classified into squamous cell carcinoma (SQC) and adenocarcinoma (ADC). Recent studies have observed marked differences on clinicopathology, chemotherapeutic responses and prognosis between lung squamous cell carcinoma and adenocarcinoma patients [3]. Notably, the overall survival time in patients with SQC is much shorter than that in patients with ADC [3, 4]. Therefore, it is of great necessity to identify the underlying molecular mechanism in SQC, which would be helpful for developing an effective therapeutic strategy against SQC.

Angiogenesis is characterized by tumor cells-induced proliferation and migration of vascular endothelial cells via varying mechanisms [5]. Targeting the proliferation and migration of vascular endothelial cells is a promising strategy for lung cancer therapy because these processes play an important role in sustaining the development of tumor growth and metastasis [6]. Vascular endothelial growth factor (VEGF) is the main mediator of the proliferation and migration of vascular endothelial cells [7]. Recent evidence suggests that VEGF directly targets tumor cells and promotes cancer growth and metastasis [8]. Recombinant VEGF is able to activate various pro-angiogenic pathways in response to hypoxia, which can promote lung cancer progression [9]. The disruption of VEGF autocrine activity in lung cancer cells has been reported to be a preventive regimen for tumor formation [10]. However, since the high-risk adverse effects of antiangiogenic therapy on squamous-cell histology are devastating, especially hemoptysis, the application of antiangiogenic therapy in SQC is limited [11–14]. Hence, further identification of molecular-targeted antiangiogenic therapy might provide a clinical benefit for SQC patients.

Long noncoding RNAs (lncRNAs) are a class of nonprotein coding RNAs with lengths of more than 200 nucleotides that are implicated in multiple biological processes, including as scaffold proteins between proteins and genes, decoys to bind proteins and enhancers to modulate the transcription of their target genes [15]. Furthermore, the function of lncRNAs as competing endogenous RNAs (ceRNAs) has received great attention as a “miRNA sponge” to disrupt miRNA-mediated degradation of target mRNAs [16], and dysregulation of miRNAs has been extensively demonstrated to contribute to tumorigenesis, progression and metastasis in numerous cancer types [17–19], including lung cancer [20, 21]. Importantly, several lines of evidence have shown that the lncRNAs mediate in tumor progression by participating in the regulation of VEGF in lung cancer [22]. Thus, strategies exploiting the anti-angiogenic role of lncRNAs may be used as therapeutic regimens in SQC.

In this study, we found that LINC00173.v1 was specifically upregulated in SQC tissues, and that high expression of LINC00173.v1 was significantly correlated with poor overall and progression-free survival in SQC patients. Gain and loss of function assays demonstrated that upregulating LINC00173.v1 promoted, while silencing LINC00173.v1 attenuated the proliferation and migration of vascular endothelial cells and tumorigenesis of SQC cells in vitro and in vivo; these effects were dependent on miR-511-5p/VEGFA axis. Importantly, administering antisense oligonucleotides against LINC00173.v1 not only inhibited the tumorigenesis of SQC cells, but also sensitized the chemotherapeutic response of SQC cells to cisplatin *in vivo*. Our results further revealed that squamous cell carcinoma-specific factor ΔNp63α was involved in LINC00173.v1 overexpression in SQC. Taken together, our results deepen the understanding of the functional role of LINC00173.v1 in the proliferation and migration of vascular endothelial cells and tumorigenesis of SQC, which will facilitate the development of anti-tumor therapeutics targeting the proliferation and migration of vascular endothelial cells in SQC.

## Methods and materials

### Cells and cell culture

Human umbilical vein endothelial cell line (HUVEC) was purchased from PromoCell (Germany). Normal lung bronchial epithelial cell line BEAS-2B, human lung adenocarcinoma cell lines Calu-3, NCI-H1395, NCI-H1975, NCI-H2228, NCI-H2347, lung squamous carcinoma cell lines NCI-H520, other non-small cell lung cancer cell lines A549, Calu-1, NCI-H292, NCI-H460, NCI-H596, NCI-H661, NCI-H1299, small cell lung cancer cell lines NCI-H209, NCI-H446, normal lung fibroblast cells MRC-5 and WI-38, normal human embryonic fibroblast cells HFL1 were obtained from Procell (Wuhan, China). Lung squamous carcinoma cell lines NCI-H226, SK-MES-1 were obtained from Shanghai Chinese Academy of Sciences cell bank (Shanghai, China). HUVEC was cultured in Endothelial Cell Growth Medium BulletKit (EGM, Lonza, Switzerland). BEAS-2B was grown in Bronchial Epithelial Cell Growth Medium BulletKit (BEGM, Lonza, Switzerland). Calu-3, SK-MES-1, MRC-5 and WI-38 were cultured in Eagle’s Minimum Essential Medium (MEM, Gibco, USA). NCI-H1395, NCI-H1975, NCI-H2228, NCI-H2347, NCI-H226, NCI-H520, NCI-H209, NCI-H292, NCI-H446, NCI-H460, NCI-H596, NCI-H661 and NCI-H1299 were maintained in RPMI-1640 (Gibco, USA). A549 and HFL1 were grown in F12K Medium (Gibco, USA), and Calu-1 was grown in McCoy’s 5a Medium (Gibco, USA). All cell lines, except for HUVEC and BEAS-2B, were supplemented with 10% fetal bovine serum (Gibco, USA). All cell lines were authenticated using short tandem repeat (STR) profiling by Hybribio Limited (China). Cells were incubated at 37 °C in a humidified atmosphere with 5% CO_2_.

### Patients and clinical samples

The 10 paired lung ADC tissues, 10 paired lung SQC tissues and the 20 corresponding matched adjacent tumor normal tissues were obtained during surgery and the clinicopathological features of the patients are summarized in Supplemental Table 1. A total of 439 frozen section and archived lung samples, including 43 benign lung disease lesions, 248 SQC tissues, 122 ADC tissues and 26 other subtypes of lung cancer, were obtained during surgery or needle biopsy. The clinicopathological features of the 43 patients with benign lung disease are summarized in Supplemental Table 2 and the 396 patients with NSCLC are summarized in Supplemental Table 3. All tissues were collected from the Affiliated Jiangmen Hospital of Sun Yat-sen University (Guangdong, China) between January 2008 and December 2018. Patients were diagnosed based on clinical and pathological evidence, and the specimens were immediately snap-frozen, liquid nitrogen tanks stored, or 6.0 μm frozen section, − 86 °C refrigerator stored. For the use of these clinical materials for research purposes, prior patients’ consents and approval from the Institutional Research Ethics Committee of the Affiliated Jiangmen Hospital of Sun Yat-sen University were obtained (Approval number: 2019–010) The proportions of tumor vs. non-tumor in hematoxylin and eosin (H&E) staining tissue samples were evaluated by the two independent professional pathologists. All clinical lung cancer tissue samples analyzed by RT-qPCR in this study with the tumor proportions exceeding 75% were used for further analysis.

### In situ hybridization (ISH) and immunohistochemistry (IHC)

The ISH and scoring LINC00173.v1 expression were performed as previously described [18]. Briefly, the slides of frozen section were digested with proteinase K (20 μg/ml, Sangon Biotech, China) for 10 min at 37 °C, fixed in 4% paraformaldehyde for 10 min at room temperature, and then dehydrated by immersion in an ethanol gradient and air dried, slides were pre-hybridized by Hybridization Denatured Buffer (Exon Biotechnology, China) at 77 °C for 30 min. The biotin-labeled LINC00173.v1 probe (the sequence of LINC00173 probe was 5′- ACAAGCTGTGACAGGTGATCA − 3′), which was designed and synthesized by Exon Biotechnology (China), denatured in hybridization buffer at 77 °C for 5 min and chilled on ice immediately. Each slide was covered with 20–50 μl diluted probe and incubated in a humidified hybridization chamber at 37 °C overnight. Slides were washed twice in 2 × SSC/0.1% Tween-20 at 37 °C for 15 min. Then the slides were blocked with blocking buffer (PBS/0.05% Tween-20 with 1% BSA) at room temperature for 30 min, and the samples were incubated with HRP-conjugated Streptavidin (Proteintech, China) at 37 °C for 1 h. After washing three times in PBS/0.05% Tween-20, the slides were stained with 3,3′-Diaminobenzidine (DAB) Enhanced Liquid Substrate System (Sigma-Aldrich, USA) for 3 min, counterstained with hematoxylin for 1 min, dehydrated by immersion in in an ethanol gradient and sealed by coverslip.

Staining index (SI) given by the two independent investigators were averaged for further comparative evaluation of LINC00173.v1 expression. Tumor cell proportion was scored as follows: 0 (no positive tumor cells); 1 (< 10% positive tumor cells); 2 (10–35% positive tumor cells); 3 (35–70% positive tumor cells) and 4 (> 70% positive tumor cells). Staining intensity was graded according to the following criteria: 0 (no staining); 1 (weak staining, light yellow); 2 (moderate staining, yellow brown) and 3 (strong staining, brown). SI was calculated as the product of staining intensity score and the proportion of positive tumor cells. Based on this method of assessment, LINC00173.v1 expression in lung tumor samples was evaluated by the SI, with scores of 0, 1, 2, 3, 4, 6, 8, 9 or 12. SI score 4 was the median of all sample tissues SI. High and low expression of LINC00173.v1 were stratified by the follow criteria: The SI ≤4 was used to define tumors with low expression of LINC00173.v1, and SI score of > 6 as tumors with high expression of LINC00173.v1.

The immunohistochemistry procedure of p40 (ΔNp63α) expression were performed as previously described [23]. Briefly, the slides of formalin-fixed paraffin-embedded section were antigen-retrieved in TE (pH 9.0) buffer 10 min by microwave heating, blocked by hydrogen peroxide and goat serum respectively, and incubated overnight at 4 °C in a humidified chamber with the anti-p40 antibody (Zsbio, China) diluted 1:100 in Antibody Diluent (Abcam, USA). After incubation, slides were washed in TBS/0.05% Tween-20, incubated with biotin-conjugated secondary antibody (Proteintech, China) and peroxidase-conjugated streptavidin (Proteintech, China) 30 min at 37 °C respectively, stained by 3,3′-Diaminobenzidine (DAB) Enhanced Liquid Substrate System (Sigma-Aldrich, USA). According to the guidelines [24], any staining of tumour cells was evaluated as positive of p40 expression.

### Plasmid, small interfering RNA and transfection

The human full-length LINC00173.v1 was PCR-amplified from cDNA and cloned into the Ubi-MCS-3FLAG-SV40-neomycin lentiviral vector (GV350, Genechem, China). The short hairpin RNA (shRNA) for human LINC00173.v1 was cloned into a hU6-MCS-CBh-gcGFP-IRES-puromycin lentiviral vector (GV493, Genechem, China). The binding site activity of LINC00173.v1 and 3’UTR of human VEGFA.v6 were PCR-amplified from genomic DNA and cloned into pmirGLO vector (Promega, USA). The list of primers used in clone reactions was presented in Supplemental Table 4. Agomir-511-5p, antagomir-511-5p and their relative controls were synthesized and purified by RiboBio (Guangzhou, China). Transfection of plasmids was performed using Lipofectamine 3000 reagent (Invitrogen, USA) according to the manufacturer’s instructions. Stable cell lines expressing LINC00173.v1, shLINC00173.v1#1or shLINC00173.v1#2 were generated by filtered-lentivirus infection using HEK293T cells (Guangzhou Jet Bio-Filtration Co., Ltd.), and selected with 0.5 mg/L puromycin (Sigma-Aldrich, USA) for 10 days, as described previously [23].

### Human umbilical vein endothelial cells (HUVECs) tube formation assay

Precooled Matrigel (Corning, USA) was added into wells of a 24-well plate and polymerized for 30 min at 37 °C. HUVECs (2 × 10^4) suspended in 200 μl of conditional medium were added to each well and incubated at 37 °C in 5% CO_2_ for 6–12 h. The images of tube structure were captured under a 100× bright-field microscope, and quantification of tube formation was measured by the mesh and length of the completed tubes by Image View 3.7 (Jingtong, China).

### Migration assay

Cells (4 × 10^4) were seeded into the upper compartment of the 24-well Transwell permeable chambers (Corning, USA), and the lower chamber of the Transwell was filled with completed media supplemented with 10% FBS as a chemoattractant. After incubation for 24 h, cells migrated to the bottom side of the chamber were fixed with stationary solution (methanol: acetic acid = 3:1), stained with crystal violet, and photographed and quantified by counting in 5 random fields.

### Chicken chorioallantoic membrane (CAM) assay

CAM assay was performed at the eighth day of fertilized chicken eggs. A 1.0 cm diameter hole was opened on the air sac of eggshell and the surface of the dermic sheet on the floor of the air sac was removed to expose the CAM. A 0.5 cm diameter filter paper was placed on the top of CAM, 100 μl of freshly harvested conditional medium from the indicated lung cancer cells was carefully added onto the center of the paper and sealed with medical breathable adhesive tape. Then, the eggs were incubated at 37.8 °C under 60 to 80% humidity for 5 days. Following fixation with stationary solution (methanol: acetone = 1:1) for 15 min, CAMs were cut out and harvested. The images of CAMs were taken using a digital camera (Canon, Japan) and analyzing by Image View 3.7 (Jingtong, China). The effect of conditional media for proliferation and migration of vascular endothelial cells was evaluated by the number and length of 2nd and 3rd vessels compared with control groups as previously described [25–27].

### Animal study

Xenograft tumor experiments were approved by the Institutional Animal Care and Use Committee of Guangdong Medical University (Approval number: GDY1902211). At least 6 or 8 BALB/c-nu mice (4–6 wk. old; 18–20 g) per group were used to ensure the adequate power and each mouse with different weight was randomly allocated. For transplantation tumor assay, 1–4 × 10^6 cells in 100 μl PBS were inoculated subcutaneously into the inguinal folds of the nude mice respectively. After 7 days, NCI-H1975–LINC00173.v1-inoculated mice were randomly divided into three groups, and treated intraperitoneally with bevacizumab (10 mg/kg), agomir-511-5p (10 μmol) or vehicle control (PBS) once a week for 4 weeks, NCI-H520-sh LINC00173#1-inoculated mice were treated with rhVEGFA (2.5 μg, R&D Systems, USA), antogomir-511-5p (10 μmol) or vehicle control respectively once a week for 4 weeks.

For tail vein injection, 1–2 × 10^6 cells in 100 μl PBS were injected into the lateral tail vein of BALB/c-nu mice (4–6 wk. old; 18–20 g). After 7 days of inoculation, mice were treated intraperitoneally with cisplatin (CDDP; 5 mg/kg) twice per week for 4 weeks. Antisense oligonucleotide (ASO) targeting LINC00173.v1 was designed and used to evaluate the pathophysiological function of endogenous LINC00173.v1 in the tumorigenesis. Mice were injected intraperitoneally with low- and high- dose of LINC00173.v1 antisense LNA™ GapmeRs (5 mg/kg, ASO-LD; 25 mg/kg, ASO-HD; Exiqon, Denmark) or negative control (PBS) weekly for 4 weeks. Mice were monitored twice weekly and were sacrificed by cervical dislocation dependent on survival time. Tumor volume was determined using an external caliper and calculated using the eq. (L × W^2)/2, and tumors were excised, weighed and partly stored in liquid nitrogen tanks. All the tissues were finally paraffin embedded and subjected to immunohistochemistry (IHC) or hematoxylin and eosin (H&E) staining following the same protocol as clinical samples. IHC was performed using anti-CD31 Monoclonal Antibody (Cell Signal Technology, USA). The number of tumor cell per mm^2^ was calculated as previous described [28]. Briefly, the tumor cell number was evaluated in 9 random fields (mm^2^) of the H&E tissues under 20X magnification using Precipoint M8 Digital Microscopy (Germany). Then, the cell number in each field was added up to be used for analysis.

### High throughput data processing and visualization

The clinical profile of lung cancer dataset, RNA sequencing profile were downloaded from The Cancer Genome Atlas (TCGA; https://tcga-data.nci.nih.gov/tcga/) and the analysis for RNA sequencing profile was used Excel 2016 (Microsoft Inc., USA) and GraphPad 5 (GraphPad Software Inc., USA) software. The 17 RNA sequencing profiles of non-small cell lung cancer based on Affymetrix U133 Plus2.0 microarray were downloaded from ArrayExpress (http://www.ebi.ac.uk/arrayexpress/). Integrated analysis of all data collected from ArrayExpress using YuGene program on R3.4.2 software (https://www.r-project.org/) was described previously [23, 29], including 340 normal lung tissues and 1788 lung cancer tissues, and the integrative expression profile of lung cancer was named for AE-meta dataset. The expression values were normalized by z-score, and performed on heat map by MeV4.9 software (http://mev.tm4.org/).

Gene Set Enrichment Analysis (GSEA) was performed with the RNA sequencing profile of lung cancer from TCGA as the expression dataset. The high and low expression levels of LINC00173 were stratified by the medium expression level of LINC00173 in all lung cancer tissues. GSEA signatures was performed by Molecular Signatures Database v6.2, and all processing parameters as the default in GSEA3.0 software (Broad Institute, USA, http://www.gsea-msigdb.org/gsea/index.jsp).

### Statistical study

All values are presented as means ± standard deviation (SD). Significant differences were determined using GraphPad 5 software. Student’s t-test was used to determine statistical differences between two groups. One-way ANOVA was used to determine statistical differences between multiple testing, and post test combined with Tukey test used to compare all pairs of groups. The chi-square test or fisher’s test was used to analyze the relationship between LINC00173.v1 expression and clinicopathological characteristics. Survival curves were plotted using the Kaplan-Meier method and compared by log-rank test. *P* < 0.05 was considered significant. All the repetitive experiments were repeated three times.

## Results

### LINC00173.v1 is specifically overexpressed in lung squamous cell carcinoma

**Fig. 1:**
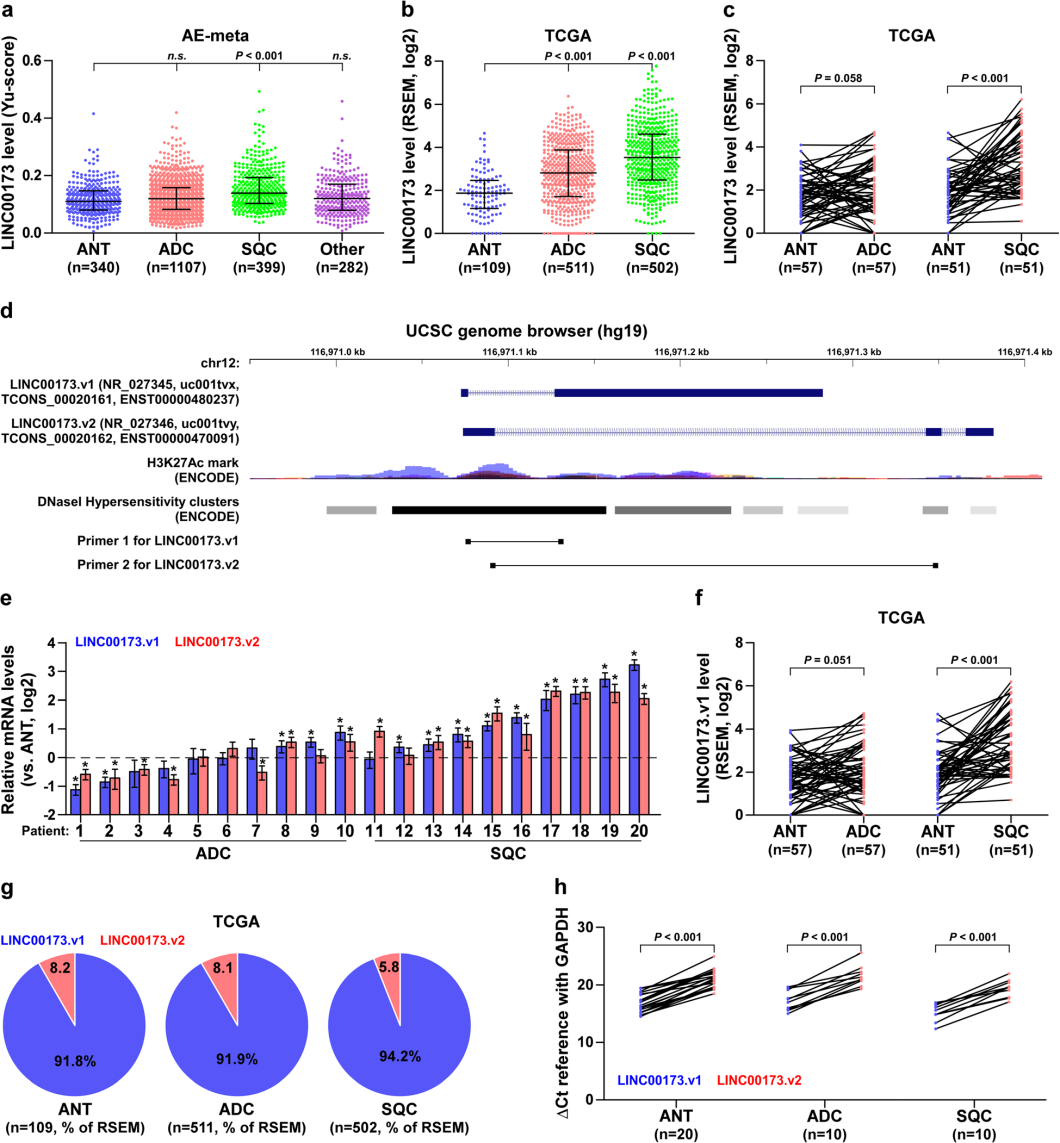
LINC00173.v1 is specifically overexpressed in lung squamous cell carcinoma. **a** Expression level of LINC00173 in 340 adjacent normal tissues (ANT), 1107 adenocarcinoma cell carcinoma (ADC), 399 squamous cell carcinoma (SQC) and 282 other subtypes of lung cancer, including Adenosquamous carcinoma (ASC), Large cell neuroendocrine carcinoma (LCNE), Undifferentiated carcinoma (UDC), Epidermoid carcinoma (EPC), Sarcomatoid carcinoma (SARC), Pleomorphic carcinoma (PMC), and Non-specific types of NSCLC in AE-meta. Each bar represents the median values ± quartile values. *P* value was determined by one-way ANOVA test. *n.s.* indicates no significance. **b** Expression level of LINC00173 in 109 ANT, 511 ADC and 502 SQC in TCGA. Each bar represents the median values ± quartile values. *P* value was determined by one-way ANOVA test. **c** Comparison of LINC00173 expression between SQC, ADC and their matched ANT in TCGA. *P < 0.05 by paired *t* test. **d** Identification of two LINC00173 transcripts and their exon sequences in UCSC genome browser. **e** RNA expression of LINC00173.v1 and LINC00173.v2 in ADC and SQC patient samples. *P < 0.05 by paired *t* test. **f** Comparison of LINC00173.v1 expression between ADC, SQC and matched ANT in TCGA. *P < 0.05 by paired *t* test. **g** RSEM percentages of LINC00173.v1 and LINC00173.v2 based on their reads in lung cancer dataset from TCGA. **h** ΔCt references of LINC00173.v1 and LINC00173.v2 in lung cancer tissues and ANT in real-time PCR analysis relative to GAPDH. *P < 0.05 by paired *t* test

To discern the lung SQC-specific relevant lncRNAs, we first analyzed our previously integrative data profile of lung cancer based on the Affymetrix U133 Plus2.0 microarray (AE-meta) [30], and found that LINC00173, one of the rarely studied lncRNAs, was specifically overexpressed in SQC compared with lung ADC and other lung cancer subtypes (Fig. 1a). Although TCGA dataset analysis showed that LINC00173 expression was upregulated in both SQC and ADC tissues (Fig. 1b), overexpression of LINC00173 was only observed in paired SQC tissues compared to their matched adjacent normal tissues (ANT) (Fig. 1c). Through analyzing the UCSC genome browser, two LINC00173 transcripts with totally different exon sequences were identified (LINC00173.v1 and LINC00173.v2) (Fig. 1d), expression levels of both LINC00173.v1 and LINC00173.v2 were significantly upregulated in SQC compared with to ADC (Fig. 1e), which was further supported by TCGA analysis (Fig. 1f and Supplemental Figure 1a-c). Consistently, LINC00173.v1 and LINC00173.v2 were dramatically upregulated in SQC cell lines compared with normal lung bronchial epithelial cell line BEAS-2B, normal lung fibroblast cell lines MRC-5 and WI-38, normal human embryonic fibroblast cell line HFL1, ADC cell lines, NSCLC cell lines (other non-ADC or SQC NSCLC cell types) and small cell lung cancer (SCLC) cell lines (Supplemental Figure 1d). To determine the primary isoform of LINC00173 in lung cancer, we further analyzed the RSEM percentages of LINC00173.v1 and LINC00173.v2 based on their reads in the lung cancer dataset from TCGA, and found that the RSEM percentage of LINC00173.v1 was higher than that of LINC00173.v2 in ANT (91.8%), ADC (91.9%) and SQC (94.2%) (Fig. 1g). Quantitatively, real-time PCR analysis further revealed that ΔCt references relative to GAPDH of LINC00173.v1 were differentially lower than those of LINC00173.v2 in lung cancer tissues and cell lines respectively (Fig. 1h and Supplemental Figure 1e). Collectively, these findings indicate that LINC00173.v1 is primarily expressed in lung cancer tissues and is specifically upregulated in SQC tissues.

### Overexpression of LINC00173.v1 is related to relapse and metastasis in SQC

**Fig. 2:**
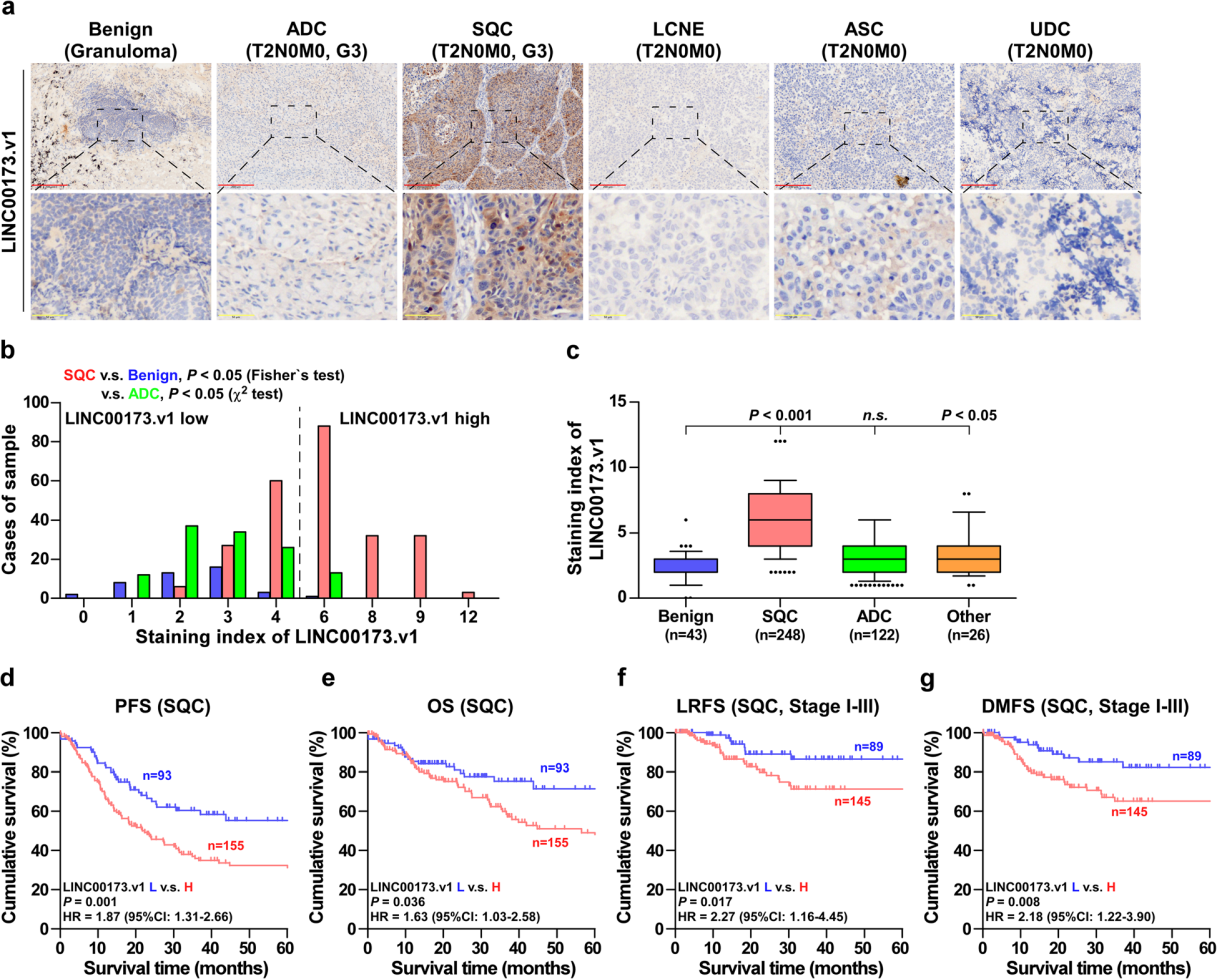
Overexpression of LINC00173.v1 predicts early relapse and metastasis in SQC**. a** Representative sections of LINC00173.v1 in 43 benign lung tissues (eg. granuloma), 248 SQC tissues, 122 ADC tissues and 26 other subtypes of lung cancer including LCNE, ASC, and UDC, using in situ hybridization. Scale bars of 100× magnification, 200 μm and 400× magnification, 50 μm. **b** The number of 43 benign lung tissues, 248 SQC tissues and 122 ADC tissues stratified by staining index of LINC00173.v1. *P* value was determined by Fisher’s test or χ^2^ test. **c** Staining index of LINC00173.v1 in 43 benign lung granuloma, 248 SQC tissues, 122 ADC tissues and 26 other subtypes of lung cancer tissues. Each bar represents the median values ± quartile values. *P* value was determined by one-way ANOVA test. *n.s.* indicates no significance. **d** to **g** Kaplan–Meier analysis of progression-free survivals (PFS) (**d**), overall survival (OS) (**e**), local relapse-free survival (LRFS) (**f**) and distant metastasis-free survival (DMFS) (**g**) in SQC patients with low LINC00173.v1 expression versus high LINC00173.v1 expression. *P* value was determined by Log-rank test. HR indicates hazard ratio; 95%CI indicates 95% confidence interval

To further investigate the clinical significance of LINC00173.v1 in lung cancer, LINC00173.v1 expression levels were examined in multiple subtypes of lung cancer using ISH. As shown in Fig. 2a and b, LINC00173.v1 expression was mainly expressed in the cytoplasm, and the staining intensity of LINC00173.v1 was slightly increased in adenosquamous cell carcinoma (ASC) tissues and strongly upregulated in SQC tissues, but was hardly detectable in benign lesions, ADC, large cell neuroendocrine carcinoma (LCNE), and undifferentiated carcinoma (UDC) tissues. Notably, high expression of LINC00173.v1 was observed in 155/248 SQC tissues (62.5%) (Fig. 2b and c). Although there was no correlation between the expression levels of LINC00173.v1 and clinicopathological characteristics (Supplemental Table 7 and Supplemental Table 8), Kaplan-Meier survival analysis showed that SQC patients with high expression of LINC00173.v1 had poorer progression-free (PFS) and overall survival (OS), as well as local relapse-free survival (LRFS) and distant metastasis-free survival (DMFS) in SQC patients compared with low expression of LINC00173.v1 (Fig. 2d-g). The prognostic significance of LINC00173.v1 in multiple lung carcinoma datasets from TCGA, AE-meta and Kaplan-Meier Plotter was further analyzed. As shown in Supplemental Figure 2a-i, patients with high levels of LINC00173.v1 predicted significantly poorer relapse-free survival and distant metastasis-free survival than those with low levels of LINC00173.v1. Taken together, our results in combination with the results from several publicly available lung carcinoma datasets suggest that overexpression of LINC00173.v1 contributes to early relapse and metastasis in SQC patients.

### Silencing LINC00173.v1 represses proliferation and migration of vascular endothelial cells

To further investigate the biological function of LINC00173.v1 in lung cancer, we first constructed stable LINC00173.v1 knockdown NCI-H226 and NCI-H520 SQC cells which expressed the relatively high basal levels of LINC00173.v1 by endogenously silencing LINC00173.v1, and exogenously overexpressed LINC00173.v1 in the ADC cell line NCI-H1975 and the NSCLC cell line NCI-H292 which expressed a relatively low basal level of LINC00173.v1 via retroviral infection (Supplemental Figure 3a). The CCK-8 assay showed that neither upregulating or silencing LINC00173.v1 had significant effects on the proliferation ability of lung cancer cells (Supplemental Figure 3b). Furthermore, neither cell cycle analysis, nor colony-formation or migration ability, was influenced by the changed expression of LINC00173.v1 in lung cancer cells (Supplemental Figure 3c-e). These results suggested that the proliferation and migration ability of lung cancer cells is not affected by LINC00173.v1 in vitro.

**Fig. 3:**
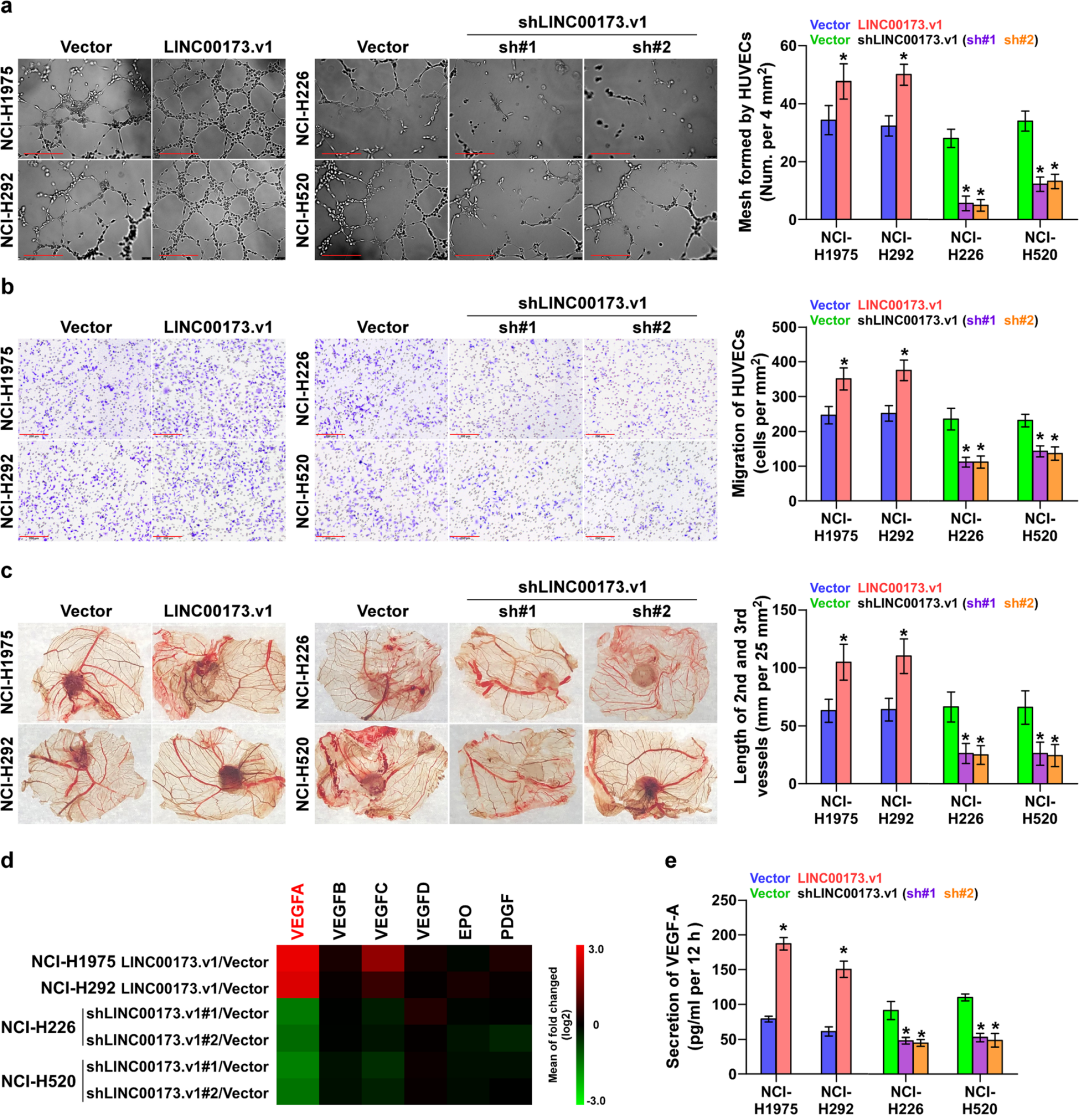
Silencing LINC00173.v1 represses proliferation and migration of vascular endothelial cells. **a** Effect of LINC00173.v1 expression in lung cancer cells on human umbilical vein endothelial cells (HUVECs) in tube formation assay. Each bar represents the mean values ± SD of three independent experiments. *P < 0.05 by unpaired *t* test or one-way ANOVA test. Scale bars, 200 μm. **b** Effect of LINC00173.v1 expression in lung cancer cells on migration ability of HUVECs in transwell assay. Each bar represents the mean values ± SD of three independent experiments. *P < 0.05 by unpaired *t* test or one-way ANOVA test. Scale bars, 200 μm. **c** Effect of LINC00173.v1 on length of the 2nd and 3rd vessels on chick embryo chorioallantoic membrane (CAM). Each bar represents the mean values ± SD of three independent experiments. *P < 0.05 by unpaired *t* test or one-way ANOVA test. **d** Real-time PCR analysis of the effect of LINC00173.v1 on expression of multiple proliferation and migration of vascular endothelial cells-associated (angiogenesis-associated) genes. Pseudo-color scale values were log2 transformed. Transcript levels were normalized by GAPDH expression. The experiment was independently performed three times. **e** Effect of LINC00173.v1 on secretion level of VEGF-A in lung cancer cells by enzyme linked immunosorbent assay (ELISA). Each bar represents the mean values ± SD of three independent experiments. *P < 0.05 by unpaired *t* test or one-way ANOVA test

Then, Gene Set Enrichment Analysis (GSEA) based on LINC00173.v1 expression data from TCGA was performed. As shown in Supplemental Figure 3f, the LINC00173.v1 expression level was strongly and positively correlated with the proliferation and migration of vascular endothelial cell-associated (angiogenesis-associated) gene signatures. Thus, we tried to explore the effect of LINC00173.v1 on the proliferation and migration of vascular endothelial cells in lung cancer cells. Tube formation assay and transwell assay revealed that overexpression of LINC00173.v1 not only promoted mesh formation by human umbilical vein endothelial cells (HUVECs), but also enhanced the migration ability of HUVECs, while silencing LINC00173.v1 yielded the opposite results (Fig. 3a and b). The angiogenic effect of LINC00173.v1 was further verified in a chick embryo chorioallantoic membrane (CAM) assay. Overexpression of LINC00173.v1 increased, while silencing LINC00173.v1 shortened the length of the 2nd and 3rd vessels of lung cancer cells cultured on CAM (Fig. 3c). Additionally, among several proliferation and migration-related genes in vascular endothelial cells, VEGF-A was obviously correlated with the variation in LINC00173.v1 expression in real-time PCR analysis (Fig. 3d and Supplemental Figure 4a). ELISA also indicated that the VEGFA expression level was significantly affected by the changed expression of LINC00173.v1 (Fig. 3e). However, upregulating LINC00173.v1 increased mRNA levels of VEGFC in NCI-H1975 and NCI-H292 cells and secretion of VEGFC in NCI-H1975 cells, but had no significant effect on secretion levels in NCI-H292 cells; silencing LINC00173.v1 did not affect mRNA expression and secretion of VEGF-C in NCI-H226 and NCI-H520 cells (Supplemental Figure 4b and c). Taken together, these findings revealed that silencing LINC00173.v1 inhibits the proliferation and migration of vascular endothelial cells by influencing the expression of VEGFA.

### Silencing LINC00173.v1 inhibits tumorigenesis in lung cancer cells

**Fig. 4:**
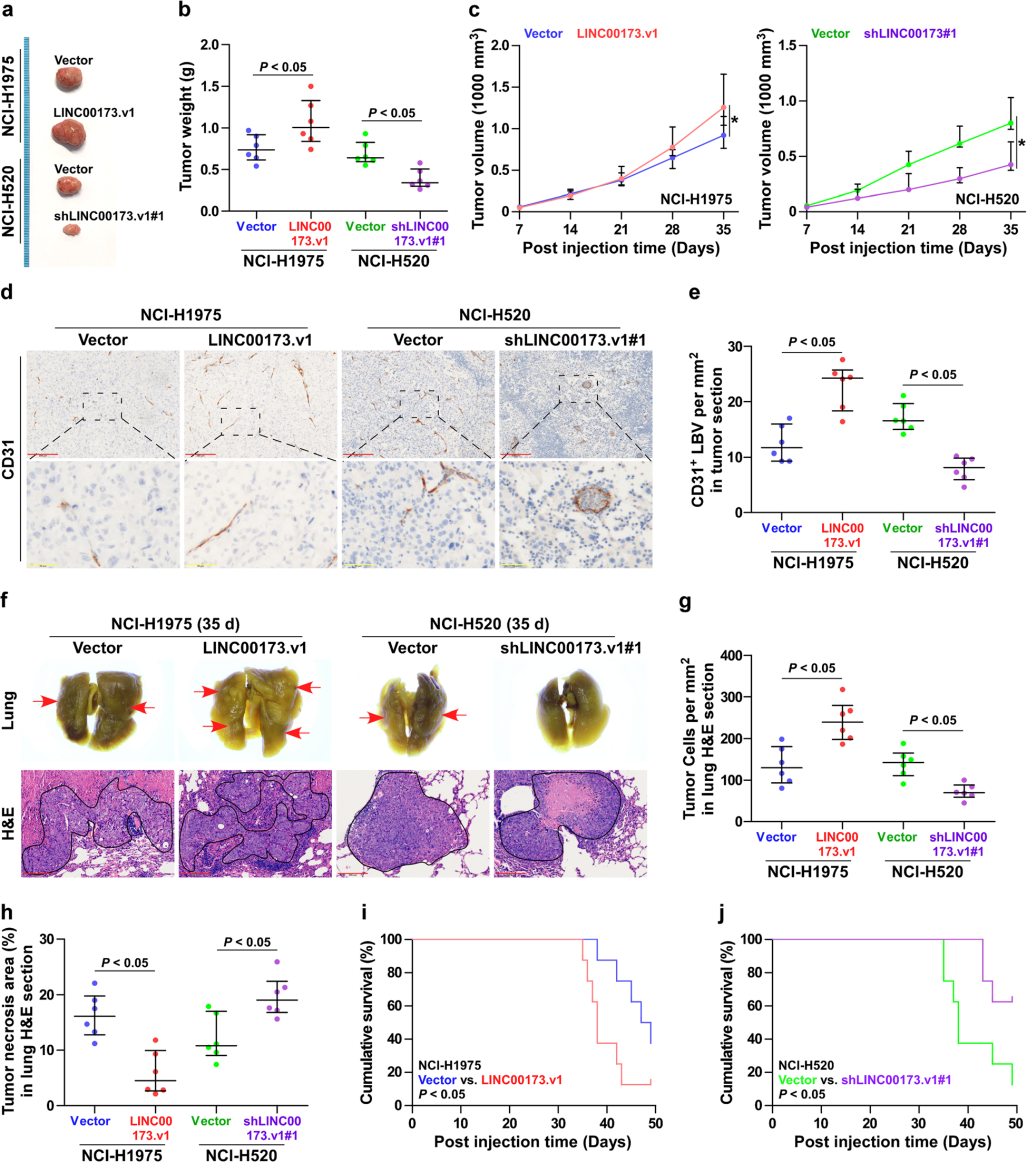
Silencing LINC00173.v1 inhibits tumorigenesis in lung cancer cells. **a** Images of the representative excised tumors from the mice at 35 days after injection with the indicated cells. **b** Average weight of excised tumors from the indicated mice (*n* = 6). Each bar represents the median values ± quartile values. *P* value was determined by unpaired *t* test. **c** Tumor volumes (*n* = 6) were measured every 7 days. Each bar represents the median values ± quartile values. *P < 0.05 by unpaired *t* test in final measurement. **d** and **e** Representative sections and CD31^+^ lymphatic or blood vessel (LBV) density in tumor tissues from the indicated mice groups (*n* = 6). after 5 weeks of cell injection. Each bar represents the median values ± quartile values. *P* value was determined by unpaired *t* test. Scale bars of 100× magnification, 200 μm and 400× magnification, 50 μm. **f** Metastatic lung tumor nests (red arrow) in the indicated mice group. Lung metastatic tumor tissues in mice were confirmed by H&E staining. Scale bars, 200 μm. **g** and **h** Tumor cell number per mm^2^ and tumor necrotic area in lung H&E section from the indicated mice groups after 5 weeks of tail veins injection. Each bar represents the median values ± quartile values. *P* value was determined by unpaired *t* test. **i** and **j** Kaplan–Meier analysis of the effect of LINC00173.v1 overexpression (**i**) and downexpression (**j**) on cumulative survival in the indicated mice groups (*n* = 8). *P* value was determined by Log-rank test

To determine the effect of LINC00173.v1 on tumorigenesis in vivo, the mice were randomly divided into 4 groups (*n* = 6 per group), in which the NCI-H1975/vector, NCI-H1975/LINC00173.v1, NCI-H520/vector and NCI-H520/LINC00173.v1sh#1 cells were subcutaneously inoculated into the inguinal folds of the mice. Five weeks after cell injection, the tumor tissues from the mice inoculated with NCI-H520/LINC00173.v1sh#1 cells, which exhibited a relatively low level of LINC00173.v1 at the end of the experiments (Supplemental Figure 4d and e) showed decreased tumor weights and volumes (Fig. 4a-c), and increased tumor necrotic area compared with those inoculated with NCI-H520/vector cells (Supplemental Figure 4f). Conversely, upregulating LINC00173.v1 yielded the opposite effect on NCI-H1975 cells (Fig. 4a-c and Supplemental Figure 4d-f). Importantly, upregulating LINC00173.v1 enhanced, while silencing LINC00173.v1 reduced lymphatic or blood vessel density (CD31^+^ LBV) in tumor tissues (Fig. 4d and e). Furthermore, CD31 expression levels in SQC tissues with high expression of LINC00173.v1 were significantly higher than those in SQC tissues with low expression of LINC00173.v1 (Supplemental Figure 3g).

The effects of LINC00173.v1 on the tumorigenic ability of lung cancer cells in the lung were further examined, where the cancer cells were injected into the mice via tail veins. As shown in Fig. 4f-j, silencing LINC00173.v1 remarkably suppressed the growth of NCI-H520 cells in the lung, as demonstrated by the reduced number of cancer cells per mm^2^, increased tumor necrotic area and prolonged cumulative survival. By contrast, upregulating LINC00173.v1 enhanced the number of cancer cells per mm^2^ (Fig. 4g), but reduced the tumor necrotic area (Fig. 4h) and cumulative survival of the mice (Fig. 4i). Notably, LINC00173.v1 dramatically affected the formation of large tumor nodules (tumor cell count > 50 each nodule) (Supplemental Figure 4g), but had no significant influence on small tumor nodules (tumor cell count < 50 each nodule) (Supplemental Figure 4h). This finding suggested that LINC00173.v1 may play a more important role in the late outgrowth of cancer cells after colonization in the lung. Finally, the LINC00173.v1 expression level was upregulated in the lung tumor tissues obtained from the mice inoculated with NCI-H1975/LINC00173.v1 cells compared with those from the mice inoculated with the vector control cells at the end of the experiments, whereas it was reduced in the lung tumor tissues from the mice inoculated with NCI-H520/LINC00173.v1sh#1 cells (Supplemental Figure 4i and j). Collectively, our findings demonstrated that silencing LINC00173.v1 inhibits the tumorigenesis of lung cancer cells in vivo.

### LINC00173.v1 functions as a competing endogenous RNA for miR-511-5p

**Fig. 5:**
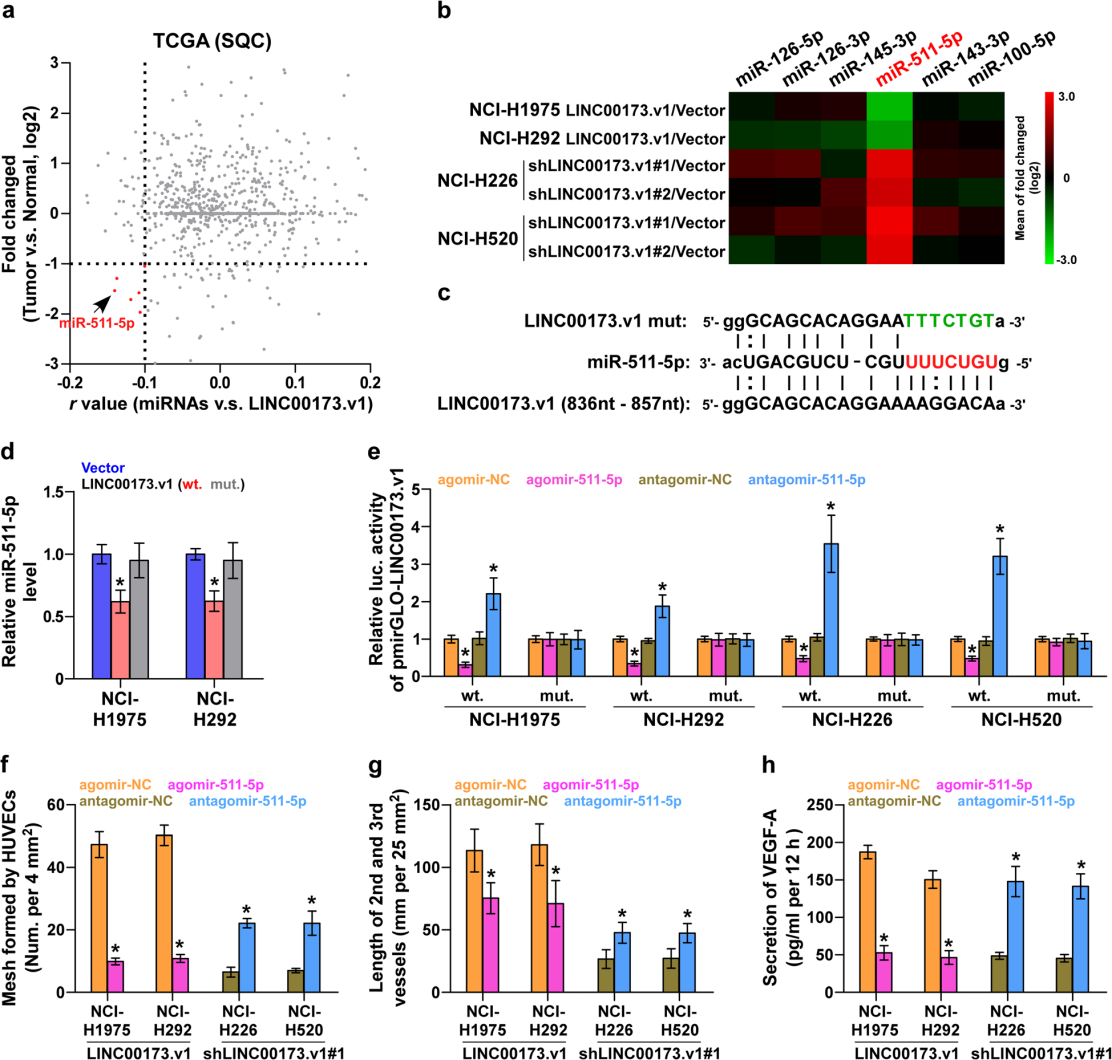
LINC00173.v1 functions as a competitive endogenous RNA for miR-511-5p. **a** Volcano plot analyzed the clinical correlation of LINC00173.v1 with all reported miRNAs in SQC dataset from TCGA (*n* = 502). The red colors represent significantly and negatively correlated miRNAs with fold change < 0.5 and *r* value < − 0.1. **b** Real-time PCR analysis of the effect of LINC00173.v1 on multiple miRNAs expression in the indicated cells. Pseudo-color scale values were log2 transformed. Transcript levels were normalized by U6 expression. The experiment was independently performed three times. **c** Potential binding sites of mutant and wild-type LINC00173.v1 on miR-511-5p. **d** Real-time PCR analysis of miR-511-5p expression compared with mutant and wild-type LINC00173.v1. Transcript levels were normalized by U6 expression. Each bar represents the mean values ± SD of three independent experiments. *P < 0.05 by one-way ANOVA test. **e** Luciferase reporter activity of the influence of miR-511-5p on LINC00173.v1. Each bar represents the mean values ± SD of three independent experiments. *P < 0.05 by one-way ANOVA test. **f** Effect of miR-511-5p on human umbilical vein endothelial cells (HUVECs) in tube formation assay in the indicated lung cancer cells. Each bar represents the mean values ± SD of three independent experiments. *P < 0.05 by unpaired *t* test. **g** Effect of miR-511-5p on length of the 2nd and 3rd vessels on chick embryo chorioallantoic membrane (CAM) in the indicated lung cancer cells. Each bar represents the mean values ± SD of three independent experiments. *P < 0.05 by unpaired *t* test. **h** Influence of miR-511-5p on secretion level of VEGF-A in the indicated lung cancer cells. Each bar represents the mean values ± SD of three independent experiments. *P < 0.05 by unpaired *t* test

By nuclear-cytoplasmic fractionation assay, we found that LINC00173.v1 was mainly expressed in the cytoplasm of four lung cancer cell lines (Supplemental Figure 5a), which was consistent with the finding that LINC00173.v1 was mainly detected in the cytoplasm in clinical lung cancer tissues via in situ hybridization as demonstrated above (Fig. 2a). Cytoplasmic lncRNAs most often function as competing endogenous RNAs (ceRNAs) to regulate downstream mRNAs by sponging miRNAs [31], suggesting that LINC00173.v1 may exert its function in lung cancer in a ceRNA manner. Then, we further analyzed the correlation of LINC00173.v1 with all reported miRNAs in the lung cancer dataset from TCGA, and found that the expression levels of six miRNAs, including miR-126-5p, miR-126-3p, miR-145-3p, miR-511-5p, miR-143-3p and miR-100-5p, were negatively correlated with LINC00173.v1, and were downregulated in SQC tissues compared with those in ANT (Fig. 5a). However, real-time qPCR analysis further revealed that only the miR-511-5p expression level was significantly affected by the changed expression of wild-type LINC00173.v1, but not the mutant LINC00173.v1 (Fig. 5b-d), which was further supported by the finding that only miR-511-5p had potential recognition sequences on LINC00173.v1, using miRanda algorithms [32] (Fig. 5c). RNA immunoprecipitation (RIP) assays demonstrated a direct association of miR-511-5p with the transcripts of LINC00173.v1 (Supplemental Figure 5b). Furthermore, a luciferase assay showed that upregulating miR-511-5p inhibited, while silencing miR-511-5p enhanced the luciferase reporter activity of LINC00173.v1, but not of the mutant LINC00173.v1 (Fig. 5e). Importantly, agomir-511-5p abolished the proliferation and migration of vascular endothelial cells induced by LINC00173.v1 overexpression in NCI-H1975 and NCI-H292 cells, whereas antagomir-511-5p reversed the inhibitory effects of LINC00173.v1 downregulation on the proliferation and migration of vascular endothelial cells in lung cancer cells (Fig. 5f-h). Therefore, these findings indicated that LINC00173.v1 promotes the proliferation and migration of vascular endothelial cells by sponging miR-511-5p as a ceRNA.

### VEGFA is a bona fide target of miR-511-5p

By analyzing miRanda algorithms [32], we found that VEGFA may be a potential target of miR-511-5p (Supplemental Figure 5c), which has been further demonstrated by the aforementioned real-time qPCR analysis (Fig. 3d). RT-qPCR analysis further revealed that upregulating miR-511-5p reduced, while silencing miR-511-5p increased the mRNA expression levels of VEGFA (Supplemental Figure 5d). The RIP assay further revealed a direct association of miR-511-5p with the VEGFA transcript (Supplemental Figure 5e), demonstrating the direct repressive effect of miR-511-5p on VEGFA. A luciferase assay showed that upregulating miR-511-5p attenuated, while silencing miR-511-5p elevated the reporter activity of the 3’UTR of VEGFA (Supplemental Figure 5f). These results indicated that VEGFA is an authentic target of miR-511-5p.

### The miR-511-5p/VEGFA axis is essential for LINC00173.v1-induced tumorigenesis in vivo

**Fig. 6:**
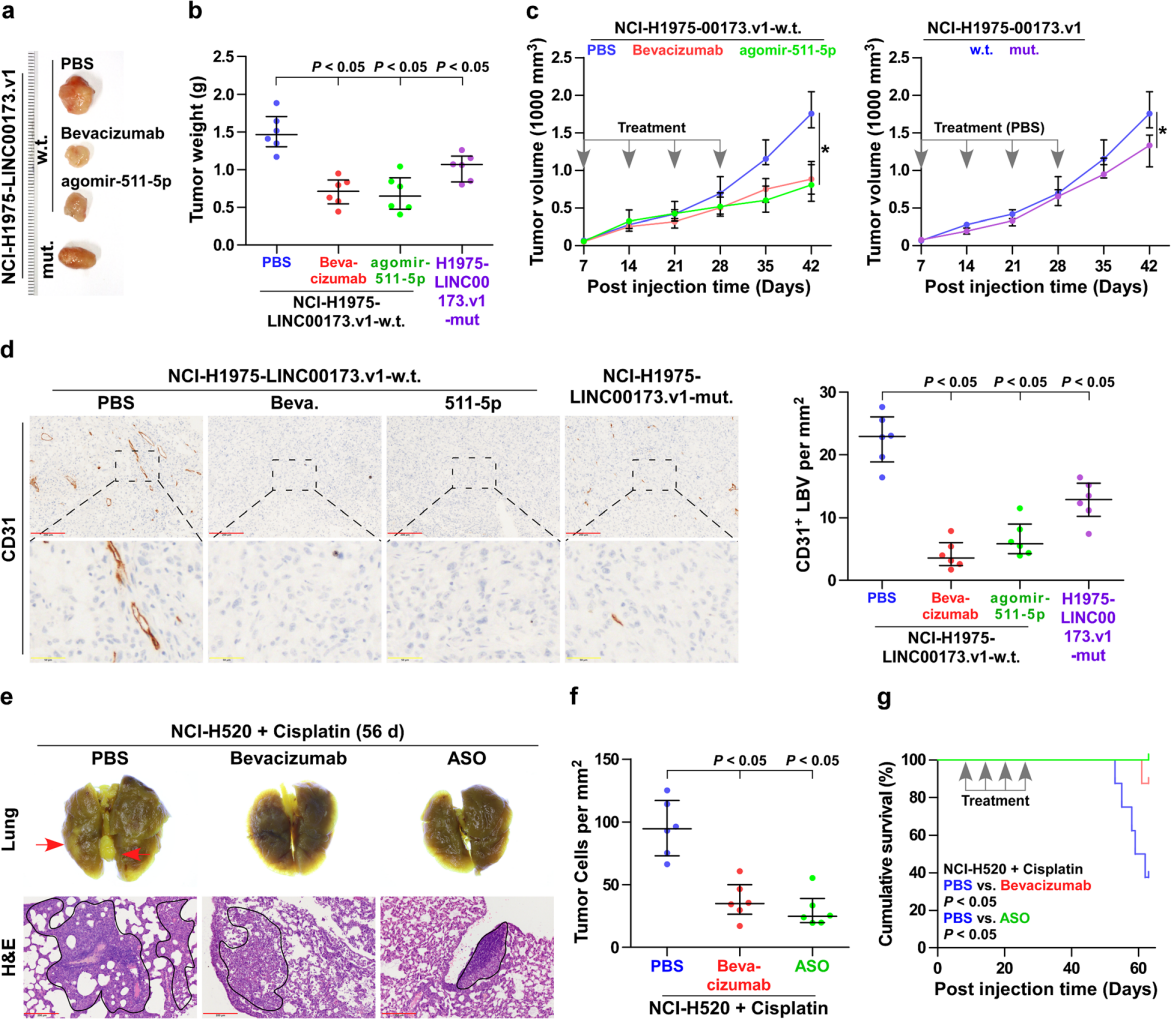
miR-511-5p/VEGFA axis is essential for LINC00173.v1-induced tumorigenesis in vivo. **a** Images of the representative excised tumors from the indicated mice at 42 days after injection. **b** Average weight of excised tumors from the indicated mice (*n* = 6). Each bar represents the median values ± quartile values. *P* value was determined by one-way ANOVA test. **c** Tumor volumes were measured every 7 days. Each bar represents the median values ±quartile values. *P* value was determined by ANOVA test in final measurement. **d** Representative sections and CD31^+^ lymphatic or blood vessel (LBV) in tumor tissues from the indicated mice groups (*n* = 6) with bevacizumab treatment, agomir-511-5p treatment and the mutant LINC00173.v1-overexpressing NCI-H1975 cells (Left panel). Density of CD31^+^ lymphatic or blood vessel (LBV) in tumor tissues from the indicated mice groups (Right panel). Each bar represents the median values ± quartile values. *P* value was determined by one-way ANOVA test. Scale bars of 100× magnification, 200 μm and 400× magnification, 50 μm. **e** Representative lung nodules (red arrow), lung H&E section and tumor cells in lung sections of therapeutic efficacy of bevacizumab and LINC00173.v1 antisense oligonucleotide (ASO) from the indicated mice groups treated with cisplatin. PBS, bevacizumab and LINC00173.v1 ASO were injected intraperitoneally once weekly for consecutive 4 weeks respectively after 1 week of cell inoculation. Scale bars, 200 μm. **f** Tumor cell number per mm^2^ in lung H&E section from the indicated mice groups after 8 weeks of tail veins injection. Each bar represents the median values ± quartile values. *P* value was determined by one-way ANOVA test. **g** Kaplan–Meier analysis of the effect of PBS, bevacizumab and LINC00173.v1 ASO on cumulative survival in the indicated mice groups (*n* = 8). *P* value was determined by Log-rank test

To further explore the functional significance of the miR-511-5p/VEGFA axis in the pro-tumor role of LINC00173.v1 in lung cancer, bevacizumab, a recombinant VEGF monoclonal antibody, was first used in a xenograft model of the mice. As shown in Fig. 6a-c, bevacizumab dramatically suppressed the growth of tumor weight and volume formed by LINC00173.v1-overexpressing NCI-H1975 cells. Likewise, it is conceivable that miR-511-5p may mediate the functional role of LINC00173.v1 in the tumorigenesis of lung cancer cells since it directly targets VEGFA. As expected, agomir-511-5p effectively inhibited the tumorigenic ability of LINC00173.v1-overexpressing NCI-H1975 cells, even reaching levels to comparable to those with bevacizumab treatment (Fig. 6a-c). On the other hand, as a ceRNA for miR-511-5p, LINC00173.v1 has been demonstrated to promote the proliferation and migration of vascular endothelial cells by sponging miR-511-5p *in vitro* (Fig. 5f-h). This supports the observation that mouse tumors formed by mutant LINC00173.v1-overexpressing NCI-H1975 cells were smaller and lighter than those formed by wild-type LINC00173.v1-overexpressing NCI-H1975 cells (Fig. 6a-c). Furthermore, the CD31^+^ LBV density in tumor tissues from the mice treated with bevacizumab, agomir-511-5p and inoculated with LINC00173.v1-overexpressing NCI-H1975 cells was differentially downregulated compared with that in the PBS group (Fig. 6d). Conversely, overexpression of VEGFA or antagomir-511-5p abrogated the inhibitory effects of LINC00173.v1 downregulation on the tumorigenesis and CD31^+^ LBV density of NCI-H520 cells (Supplemental Figure 6a-d). Taken together, our results demonstrated that LINC00173.v1 promotes the tumorigenesis of lung cancer cells via the miR-511-5p/VEGFA signaling axis.

### The therapeutic effect of LINC00173.v1 inhibition on SQC in vivo

To investigate the pathophysiological function of endogenous LINC00173.v1 in the tumorigenesis of SQC cells in vivo, an ASO targeting LINC00173.v1 was designed and used as an antagonist to inhibit endogenous LINC00173.v1 expression, as described in our previous report [33]. After 1 week of cell inoculation, low- and high-dose of ASOs were injected intraperitoneally once weekly for consecutive 4-weeks. As shown in Supplemental Figure 6e and f, inhibition of LINC00173.v1 by ASO differentially repressed the lung tumor growth, as indicated by the reduced number of cancer cells per mm^2^ and prolonged cumulative survival, determining the therapeutic efficacy of LINC00173.v1 ASOs in SQC.

As anti-angiogenic therapy is generally combined with chemotherapeutic drugs, such as cisplatin, in the clinical treatment of lung cancer [34–36], the combined effect of LINC00173.v1 ASO and cisplatin was further evaluated. As shown in Fig. 6e-g, injection of LINC00173.v1 ASO into the mice treated with cisplatin robustly promoted the therapeutic effect of cisplatin in tumor lesions derived from NCI-H520 cells, which even reached a therapeutic efficacy comparable to that of the combination of bevacizumab and cisplatin, suggesting that LINC00173.v1 inhibition might represent a valid therapeutic strategy for the treatment of SQC.

### ΔNp63α contributes to LINC00173.v1 overexpression in SQC

**Fig. 7:**
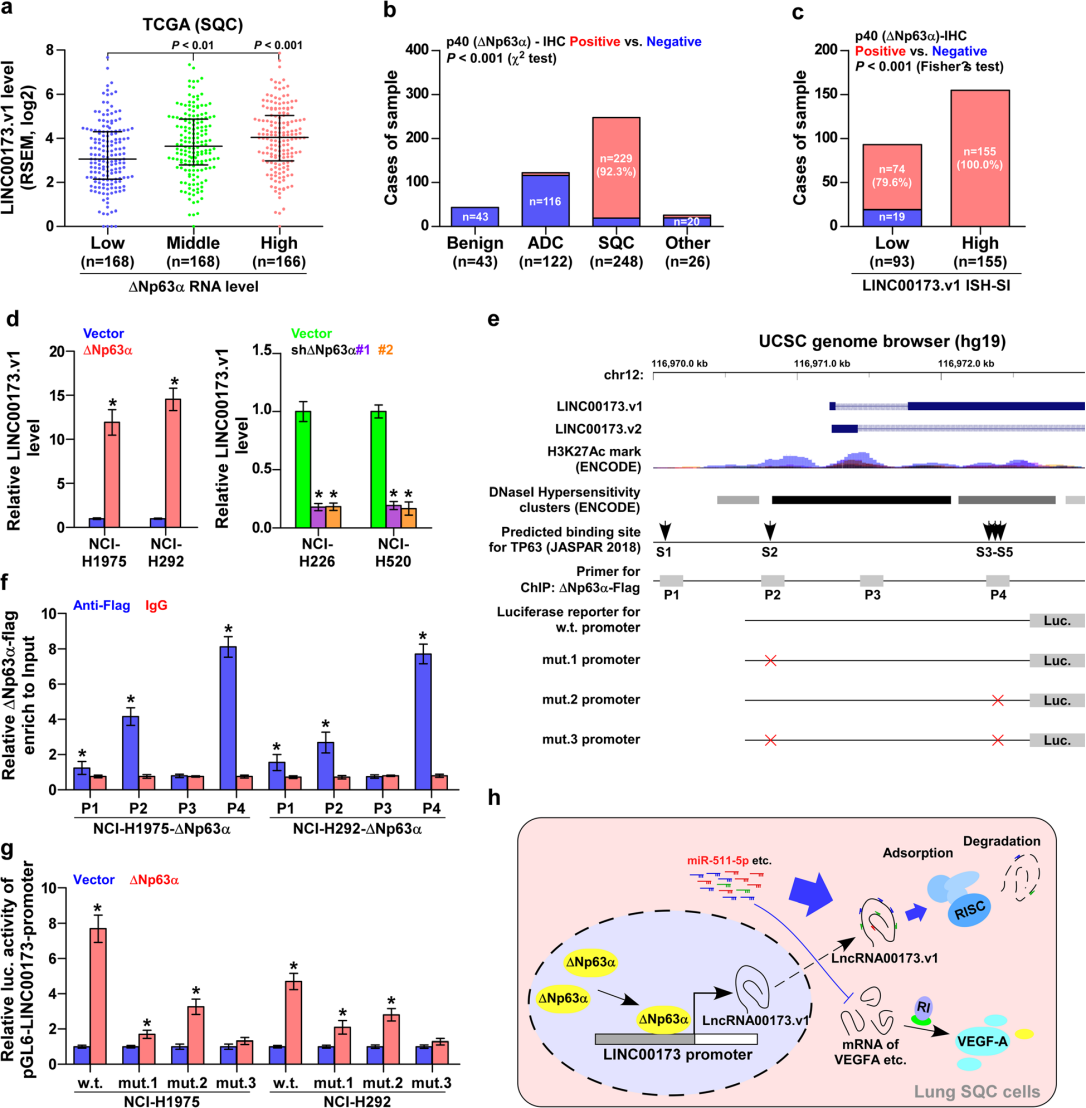
Squamous cell carcinoma-specific factor ΔNp63α contributes to LINC00173.v1 overexpression in SQC. **a** LINC00173.v1 expression levels in lung cancer tissues with low (*n* = 168), middle (*n* = 168) and high (*n* = 166) ΔNp63α expression from TCGA SQC dataset. Each bar represents the median values ± quartile values. *P* value was determined by one-way ANOVA test. **b** Positive percentage of ΔNp63α staining in 43 benign lung granuloma, 248 SQC tissues, 122 ADC tissues and 26 other subtypes of NSCLC. *P* value was determined by χ^2^ test. **c** Cases of sample of immunohistochemical staining of ΔNp63α in SQC tissues stratified by low (*n* = 93) and high (*n* = 155) LINC00173.v1 expression levels. *P* value was determined by Fisher’s test. **d** Real-time PCR analysis of LINC00173.v1 expression in lung cancer cell lines with changed expression levels of ΔNp63α. Each bar represents the mean values ± SD of three independent experiments. *P < 0.05 by unpaired *t* test or one-way ANOVA test. **e** ΔNp63α-binding motifs in the putative promoter region of LINC00173.v1 in UCSC genome browser. **f** ChIP analysis of the binding sites of ΔNp63α in the promoter region of LINC00173.v1 in lung cancer cells. IgG was used as negative control. Each bar represents the mean values ± SD of three independent experiments. *P < 0.05 by unpaired *t* test. **g** Luciferase reporter activity of LINC00173.v1 promoter in ΔNp63α-overexpressing lung cancer cells. Each bar represents the mean values ± SD of three independent experiments. *P < 0.05 by unpaired *t* test. **h** Hypothetical model illustrating that squamous cell carcinoma-specific factor ΔNp63α contributes to LINC00173.v1 overexpression in SQC tissues, which further promotes proliferation and migration of vascular endothelial cells and progression of lung squamous cell carcinoma through sponging miR-511-5p to regulate VEGFA expression

Interestingly, analysis of the TCGA lung cancer dataset revealed that LINC00173.v1 expression levels were gradually increased in SQC tissues as the expression levels of squamous cell carcinoma-specific factor ΔNp63α increased from low, to medium to high (Fig. 7a). This finding supported the notion that ΔNp63α may be responsible for the specific overexpression of LINC00173.v1 in SQC tissues. First, immunohistochemical staining analysis showed that ΔNp63α was localized in the nucleus, and was highly expressed in SQC tissues (Fig. 7b and Supplemental Figure 7a), but was slightly expressed in ASC tissues (Supplemental Figure 7a). Importantly, the percentage of positive staining of ΔNp63α expression was 100% in SQC tissues with high levels of LINC00173.v1 compared with 79.6% in those with low levels of LINC00173.v1 (Fig. 7c). Real-time PCR analysis further demonstrated that upregulating ΔNp63α elevated, while silencing ΔNp63α reduced LINC00173.v1 expression (Fig. 7d). Through UCSC bioinformatics analysis and JASPAR2018 algorithms [37], we found five ΔNp63α binding motifs in the putative promoter region of LINC00173.v1 (Fig. 7e and Supplemental Figure 7b). A ChIP assay showed that ΔNp63α had high affinity for the P2 and P4 binding sites in the promoter region of LINC00173.v1 in lung cancer cells (Fig. 7f). Consistently, enhanced luciferase activity of the LINC00173.v1 promoter was observed in ΔNp63α overexpressing lung cancer cells (Fig. 7g). However, the luciferase activity of the LINC00173.v1 promoter was decreased by mutation of either P2 or P4, but was not significantly influenced by ΔNp63α when both P2 and P4 binding sites were mutated (Fig. 7g). Therefore, our results demonstrated that ΔNp63α contributes to LINC00173.v1 overexpression in SQC tissues by regulating transcription. Taken together, these findings support the notion that squamous cell carcinoma-specific factor ΔNp63α contributes to LINC00173.v1 overexpression in SQC tissues, which further promotes proliferation and migration of vascular endothelial cells and the progression of lung squamous cell carcinoma through sponging miR-511-5p to regulate VEGFA expression (Fig. 7h).

## Discussion

The primary results of the current study provide novel insights into the critical role of LINC00173.v1 in the tumorigenesis of SQC via the miR-511-5p/VEGFA axis. Here, we reported that overexpression of LINC00173.v1 caused by ΔNp63α at the transcriptional level was observed in SQC tissues, and was significantly correlated with early relapse and distant metastasis in SQC patients. Our results further revealed that LINC00173.v1 promoted the proliferation and migration of vascular endothelial cells and the tumorigenesis of SQC cells by disrupting the repressive effect of miR-511-5p on VEGFA expression by sponging miR-511-5p. Importantly, inhibition of LINC00173.v1 by ASO reinforced the therapeutic sensitivity of SQC cells to cisplatin in vivo. Therefore, our results uncover a novel mechanism by which LINC00173.v1 promotes the proliferation and migration of vascular endothelial cells and the tumorigenesis of SQC, clarifying the tumorigenic role of LINC00173.v1 in SQC.

Although lncRNAs have been widely reported to exert their biological role via interaction with DNA, RNA and protein depending on the subcellular localization of the lncRNAs within the cells [15], accumulating studies have shown that cytoplasmic lncRNAs function to relieve miRNA-mediated target mRNA degradation as ceRNAs, which further contributes to the progression and metastasis of cancers [38–40]. Similarly, dysregulation of cytoplasmic lncRNAs has been extensively implicated in recurrence and metastasis in lung cancer [41–44]. In the current study, we found that LINC00173.v1 was mainly detected in the cytoplasm of lung cancer tissues using in situ hybridization. Further investigations revealed that upregulating LINC00173.v1 increased VEGFA expression by sponging miR-511-5p. Importantly, the stimulatory effects of LINC00173.v1 overexpression on the proliferation and migration of vascular endothelial cells in vitro and the tumorigenesis of lung cancer cells in vivo were differentially abrogated by recombinant VEGF monoclonal antibody bevacizumab, agomir-511-5p and mutant LINC00173.v1. Therefore, our findings reveal the crucial role of the LINC00173.v1/miR-511-5p/VEGFA axis in SQC.

Recent studies have demonstrated that aberrant expression of lncRNAs plays an important role in the proliferation and migration of vascular endothelial cells in lung cancer. Wang et al. has reported that overexpression of lncRNA TNK2-AS1 interacted with STAT3 to facilitate proliferation and migration of vascular endothelial cells by elevating VEGFA expression [45]. lncRNA LOC100132354 has been demonstrated to promote the proliferation and migration of vascular endothelial cells by upregulating VEGFA/VEGFR2 expression in lung adenocarcinoma, which further contributed to the tumorigenesis of lung adenocarcinoma [22]. In the current study, we found that overexpression of LINC00173.v1 promoted the proliferation and migration of vascular endothelial cells of SQC cells by upregulating VEGFA as a ceRNA to sponge miR-511-5p. Importantly, upregulating LINC00173.v1 increased CD31^+^ LBV density in vivo, suggesting that proangiogenic effect of LINC00173.v1 plays a pivotal role in the tumorigenesis of SQC in vivo. Taken together, our results clarify the underlying mechanism responsible for the pro-angiogenic role of LINC00173.v1 in SQC.

Anti-VEGF therapy has been identified as a promising option for advanced non-small cell lung cancer patients [7]. However, the high risk of fatal bleeding events, including fatal pulmonary hemorrhage, it has been widely reported, limiting its application in SQC [12]. Multiple clinical trials of anti-VEGF therapies only focused on non-squamous non-small cell lung carcinoma patients [46]. Notably, lncRNAs have been reported to be involved in tumor progression through their regulation of VEGF expression in lung cancer [22]. Thus, it is necessary to identify a new molecular target for anti-VEGF therapies against squamous cell lung cancer. In this study, our results showed that overexpression of LINC00173.v1 promoted the proliferation and migration of vascular endothelial cells in vitro and increased CD31^+^ LBV density in vivo by upregulating VEGFA as a ceRNA to sponge miR-511-5p. Importantly, our results further revealed that silencing LINC00173.v1 not only inhibited the tumorigenesis of lung cancer cells, but also reinforced the chemotherapeutic sensitivity of lung cancer cells to cisplatin in vivo when an antisense oligonucleotide (ASO) targeting LINC00173.v1 was administered. Collectively, our results indicate that a strategy targeting LINC00173.v1 might serve as a valid anti-angiogenic therapeutic regimen against SQC.

Importantly, our results demonstrated that inhibition of LINC00173.v1 with antisense oligonucleotides not only reduced the tumor growth of SQC cells in vivo, but also dramatically enhanced the therapeutic sensitivity of SQC cells to cisplatin in vivo, suggesting the potential therapeutic efficacy of LINC00173.v1 as a chemotherapeutic sensitizer in SQC. This may be explained why LINC00173 was found to be highly expressed in chemoresistant small-cell lung cancer (SCLC) cell lines, and was significantly correlated with chemoresistance and progression in SCLC patients. Furthermore, LINC00173 promoted the translocation of β-catenin by upregulating Etk, GSKIP and NDRG1 expression by sponging miRNA-218 as a ceRNA, which induced the chemoresistance and growth of SCLC tumors in vivo [47]. Therefore, our results in combination with others provided experimental evidence that LINC00173 can be recognized as a potential therapeutic target in different types of lung cancer, at least in SQC and SCLC.

## Conclusions

In summary, our results demonstrate that transcriptional upregulation of LINC00173.v1 by squamous cell carcinoma-specific factor ΔNp63α increases VEGFA expression by sponging miR-511-5p, which further promotes the proliferation and migration of vascular endothelial cells and the tumorigenesis of SQC. This improved understanding of the underlying mechanisms of LINC00173.v1 in the proliferation and migration of vascular endothelial cells and tumorigenesis of SQC will facilitate the development of anti-angiogenic therapeutic strategies in SQC.

## Supplementary information

**Additional file 1: Supplement Figure 1. (a and b)** Expression level of LINC00173.v1 and LINC00173.v2 in lung cancer and adjacent normal tissues (ANT) in TCGA. Each bar represents the median values ± quartile values. *P* value was determined by one-way ANOVA test. **(c)** Comparison of LINC00173.v2 expression between SQC and ADC, and their matched ANT in TCGA. *P* value was determined by paired *t* test. **(d)** mRNA expression of LINC00173.v1 and LINC00173.v2 in normal lung bronchial epithelial cell BEAS-2B, normal lung fibroblast cells MRC-5 and WI-38, normal human embryonic fibroblast cells HFL1, ADC cell lines, NSCLC cell lines (other non-ADC or SQC NSCLC cell types) and small cell lung cancer (SCLC) cell lines. **(e)** ΔCt references of LINC00173.v1 and LINC00173.v2 in 20 lung cancer cells in real-time PCR analysis relative to GAPDH. *P* value was determined by paired *t* test.

**Additional file 2: Supplement Figure 2. (a to d)** Kaplan–Meier analysis of of progression-free survivals (PFS), overall survival (OS), local relapse-free survival (LRFS) and distant metastasis-free survival (DMFS) in SQC patients with low LINC00173.v1 expression versus high LINC00173.v1 expression from TCGA. *P* value was determined by Log-rank test. HR indicates hazard ratio; 95%CI indicates 95% confidence interval. **(e to g)** Kaplan–Meier analysis of PFS, OS and relapse-free survival (RFS) in SQC patients with low LINC00173.v1 expression versus high LINC00173.v1 expression from AE-meta. *P* value was determined by Log-rank test. HR indicates hazard ratio; 95%CI indicates 95% confidence interval. **(h and i)** Kaplan-Meier Plotter of PFS and OS in SQC patients with low LINC00173.v1 expression versus high LINC00173.v1 expression. *P* value was determined by Log-rank test. HR indicates hazard ratio; 95%CI indicates 95% confidence interval.

**Additional file 3: Supplement Figure 3.** LINC00173.v1 does not interfere proliferation and migration ability of lung cancer cells in vitro. **(a)** Real-time PCR analysis of LINC00173.v1 expression in exogenously overexpressed LINC00173.v1 in ADC cell line NCI-H1975 and NSCLC cell line NCI-H292, and LINC00173.v1-stably downexpressing NCI-H226 and NCI-H520 SQC cells lines. Each bar represents the mean values ± SD of three independent experiments. *P < 0.05 by unpaired *t* test or one-way ANOVA test. **(b)** The effect of LINC00173.v1 on proliferation of lung cancer cells was assessed by CCK-8 assay. Each bar represents the mean values ± SD of three independent experiments. *P* value was determined by unpaired *t* test or one-way ANOVA test. *n.s.* indicates no significance. **(c)** The effects of LINC00173.v1 on the cell cycle progression of lung cancer cells. Each bar represents the mean values ± SD of three independent experiments. *P* value was determined by unpaired *t* test or one-way ANOVA test. *n.s.* indicates no significance. **(d)** The influence of LINC00173.v1 on proliferation of lung cancer cells was assessed by colony formation assay. Each bar represents the mean values ± SD of three independent experiments. *P* value was determined by unpaired *t* test or one-way ANOVA test. *n.s.* indicates no significance. **(e)** The influence of LINC00173.v1 on migration ability of lung cancer cells was assessed by Transwell assay. Each bar represents the mean values ± SD of three independent experiments. *P* value was determined by unpaired *t* test or one-way ANOVA test. *n.s.* indicates no significance. Scale bars, 200 μm. **(f)** Gene Set Enrichment Analysis (GSEA) of correlation with proliferation and migration of vascular endothelial cells-associated genes signatures based on LINC00173.v1 expression data from the TCGA. **(g)** CD31^+^ lymphatic or blood vessel (LBV) density in low and high expression SQC tissues. Each bar represents the median values ± quartile values. *P* value was determined by unpaired *t* test.

**Additional file 4: Supplement Figure 4. (**a) Effect of LINC00173.v1 on mRNA levels of VEGFA in lung cancer cells by RT-PCR analysis. Each bar represents the mean values ± SD of three independent experiments. *P < 0.05 by unpaired *t* test or one-way ANOVA test. **(b)** Effect of LINC00173.v1 on mRNA levels of VEGFC in lung cancer cells by RT-PCR analysis. Each bar represents the mean values ± SD of three independent experiments. *P < 0.05 by unpaired *t* test or one-way ANOVA test. **(c)** Effect of LINC00173.v1 on secretion level of VEGF-C in lung cancer cells by enzyme linked immunosorbent assay (ELISA). Each bar represents the mean values ± SD of three independent experiments. *P < 0.05 by unpaired *t* test or one-way ANOVA test. **(d and e)** Representative images and staining index of LINC00173.v1 in tumor tissues from the indicated mice groups (*n* = 6) after 5 weeks of cell injection. Each bar represents the median values ± quartile values. *P* value was determined by unpaired *t* test. Scale bars of 100× magnification, 200 μm and 400× magnification, 50 μm. **(f)** Necrotic area in tumor tissues from the indicated mice groups after 5 weeks of cell injection. Each bar represents the median values ± quartile values. *P* value was determined by unpaired *t* test. **(g and h)** Tumor nodules of tumor cells > 50 (d) and < 50 (e) in lung sections from the indicated mice groups after 5 weeks of cell injection. Each bar represents the median values ± quartile values. *P* value was determined by unpaired *t* test. *n.s.* indicates no significance. **(i and j)** Representative sections and staining index of LINC00173.v1 in metastatic lung tumor tissues from the indicated mice groups (*n* = 6). Each bar represents the median values ± quartile values. *P* value was determined by unpaired *t* test. Scale bars of 100× magnification, 200 μm and 400× magnification, 50 μm.

**Additional file 5: Supplement Figure 5. (a)** Nuclear–cytoplasmic fractionation assays revealed that LINC00173.v1 was abundant in cytoplasm of lung cancer cells. U6 and actin were used as positive controls in nucleus and cytoplasm, respectively. Each bar represents the mean values ± SD of three independent experiments. **(b)** RNA immunoprecipitation (RIP) assay of the enrichment of LINC00173.v1 on miR-511-5p. IgG was used as negative control. Each bar represents the mean values ± SD of three independent experiments. *P < 0.05 by one-way ANOVA test. **(c)** Predicted miR-511-5p target sequence in 3’UTRs of VEGFA.v6. **(d)** RT-qPCR analysis of the effect of miR-511-5p on VEGFA.v6 in the indicated cells. Transcript levels were normalized by GAPDH expression. Each bar represents the mean values ± SD of three independent experiments. *P < 0.05 by one-way ANOVA test. **(e)** RNA immunoprecipitation (RIP) assay of the enrichment of miR-511-5p on VEGFA.v6. IgG was used as negative control. Each bar represents the mean values ± SD of three independent experiments. *P < 0.05 by ANOVA for repeated measures. **(f)** The influence of miR-511-5p on luciferase reporter activity of VEGFA.v6. Each bar represents the mean values ± SD of three independent experiments. *P < 0.05 by one-way ANOVA test.

**Additional file 6: Supplement Figure 6. (a)** Images of the representative excised tumors from the indicated mice (*n* = 6) at 42 days after injection. **(b)** Average weight of excised tumors from the indicated mice (*n* = 6). Each bar represents the median values ± quartile values. *P < 0.05 by one-way ANOVA. **(c)** Tumor volumes were measured every 7 days. Each bar represents the median values ± quartile values. *P* value was determined by ANOVA test in final measurement. **(d)** Density of CD31^+^ lymphatic or blood vessel (LBV) in tumor tissues from the indicated mice groups. Each bar represents the median values ± quartile values. *P* value was determined by ANOVA test. **(e)** Metastatic lung tumor nests in the mice groups injected with PBS, low- and high-dose LINC00173.v1 ASO Lung metastatic tumor tissues in mice were confirmed by H&E staining (Left panel). Tumor cell number per mm^2^ in lung H&E section from the indicated mice groups after 6 weeks of tail veins injection (Right panel). Each bar represents the median values ± quartile values. *P* value was determined by ANOVA test. Scale bars, 200. **(f)** Kaplan–Meier analysis of the effect of PBS, low- and high-dose LINC00173.v1 ASO on cumulative survival in the indicated mice groups (*n* = 8). *P* value was determined by Log-rank test.

**Additional file 7: Supplement Figure 7.** Squamous cell carcinoma-specific factor ΔNp63α contributes to LINC00173.v1 overexpression in SQC **(a)** Representative sections of ΔNp63α in 43 benign lung tissues (eg. granuloma), 248 SQC tissues, 122 ADC tissues and 26 other subtypes of lung cancer including LCNE, ASC, and UDC, using immunohistochemical staining (IHC). Scale bars of 100× magnification, 200 μm and 400× magnification, 50 μm. **(b)** The putative binding sites of ΔNp63α in LINC00173 promoters by JASPAR.

**Additional file 8: Supplemental Table 1.** The basic information of 20 NSCLC patients for LINC00173.v1 RT-qPCR analysis. **Supplemental Table 2.** The basic information of 43 patients with benign lung disease for LINC00173.v1 in situ hybridization analysis. **Supplemental Table 3.** The basic information of 396 patients with NSCLC for LINC00173.v1 in situ hybridization analysis. **Supplemental Table 4.** A list of primers used in the reactions for clone PCR. **Supplemental Table 5.** A list of primers used in the reactions for RT-qPCR. **Supplemental Table 6.** A list of primers used in the reactions for CHIP assay. **Supplemental Table 7.** The relationship between LINC00173.v1 expression level and clinical pathological characteristics in 396 patients with NSCLC. **Supplemental Table 8.** The relationship between LINC00173.v1 expression level and clinical pathological characteristics in 248 patients with lung SQC.

## Data Availability

The datasets generated and analysed during the current study are available in the TCGA (https:// https://cancergenome.nih.gov/) and ArrayExpress (http://www.ebi.ac.uk/arrayexpress/). Gene Set Enrichment Analysis (GSEA) was performed using GSEA 3.0 (http://www.gsea-msigdb.org/gsea/index.jsp).) and gene set was performed by Molecular Signatures Database v5.2 (http://software.broadinstitute.org/gsea/msigdb). Integrated analysis of all data was processing in R3.4.2 software (https://www.r-project.org/). Heat map was performed by MeV4.9 software (http://mev.tm4.org/).

## Abbreviations

ADC: Adenocarcinoma
ANT: Adjacent normal tissues
ASC: Adenosquamous cell carcinoma
ASO: Antisense oligonucleotide
CAM: Chicken chorioallantoic membrane
ceRNA: competitive endogenous RNAs
cDNA: complementary DNA
ChIP: Chromatin immunoprecipitation
CNV: Copy number variation
DAB: Diaminobenzidine
DMFS: Distant metastasis-free survival
ELISA: Enzyme-linked immunosorbent assay
GAPDH: Glyceraldehyde-3-phosphate dehydrogenase
GSEA: Gene Set Enrichment Analysis
H&E: Hematoxylin and eosin
HUVEC: Human umbilical vein endothelial cell line
IHC: Immunohistochemistry
ISH: In situ hybridization
LCNE: Large cell neuroendocrine carcinoma
lncRNAs: long noncoding RNAs
LRFS: Local relapse-free survival
mRNA: messenger RNA
NSCLC: Non-small cell lung cancer
RIP: RNA immunoprecipitation
SCLC: Small cell lung cancer
SD: Standard deviation
shRNA: short hairpin RNA
SI: Staining index
SQC: Squamous cell carcinoma
STR: Short tandem repeat
TCGA: The Cancer Genome Atlas
VEGF: Vascular endothelial growth factor
