# Supplementary figures and images for: LINC00173.v1 promotes angiogenesis and progression of lung squamous cell carcinoma by sponging miR-511-5p to regulate VEGFA expression
Source: Mol Cancer. 2020 May 30;19:98. doi: 10.1186/s12943-020-01217-2 (PMC7260858; doi:10.1186/s12943-020-01217-2)

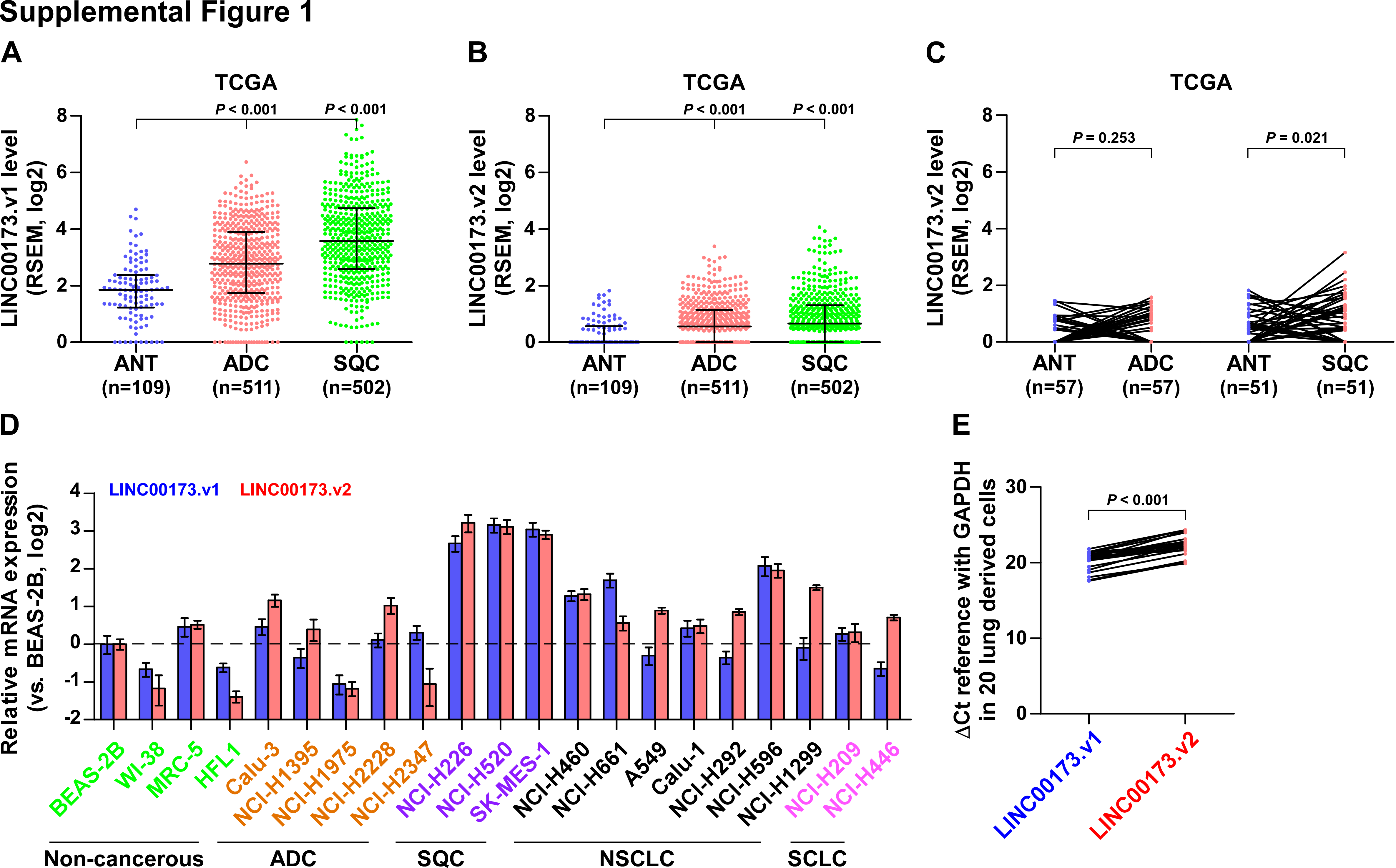

Supplement: Supplementary file 1 — Additional file 1: Supplement Figure 1. (a and b) Expression level of LINC00173.v1 and LINC00173.v2 in lung cancer and adjacent normal tissues (ANT) in TCGA. Each bar represents the median values ± quartile values. P value was determined by one-way ANOVA test. (c) Comparison of LINC00173.v2 expression between SQC and ADC, and their matched ANT in TCGA. P value was determined by paired t test. (d) mRNA expression of LINC00173.v1 and LINC00173.v2 in normal lung bronchial epithelial cell BEAS-2B, normal lung fibroblast cells MRC-5 and WI-38, normal human embryonic fibroblast cells HFL1, ADC cell lines, NSCLC cell lines (other non-ADC or SQC NSCLC cell types) and small cell lung cancer (SCLC) cell lines. (e) ΔCt references of LINC00173.v1 and LINC00173.v2 in 20 lung cancer cells in real-time PCR analysis relative to GAPDH. P value was determined by paired t test. [file 12943_2020_1217_MOESM1_ESM.tif]

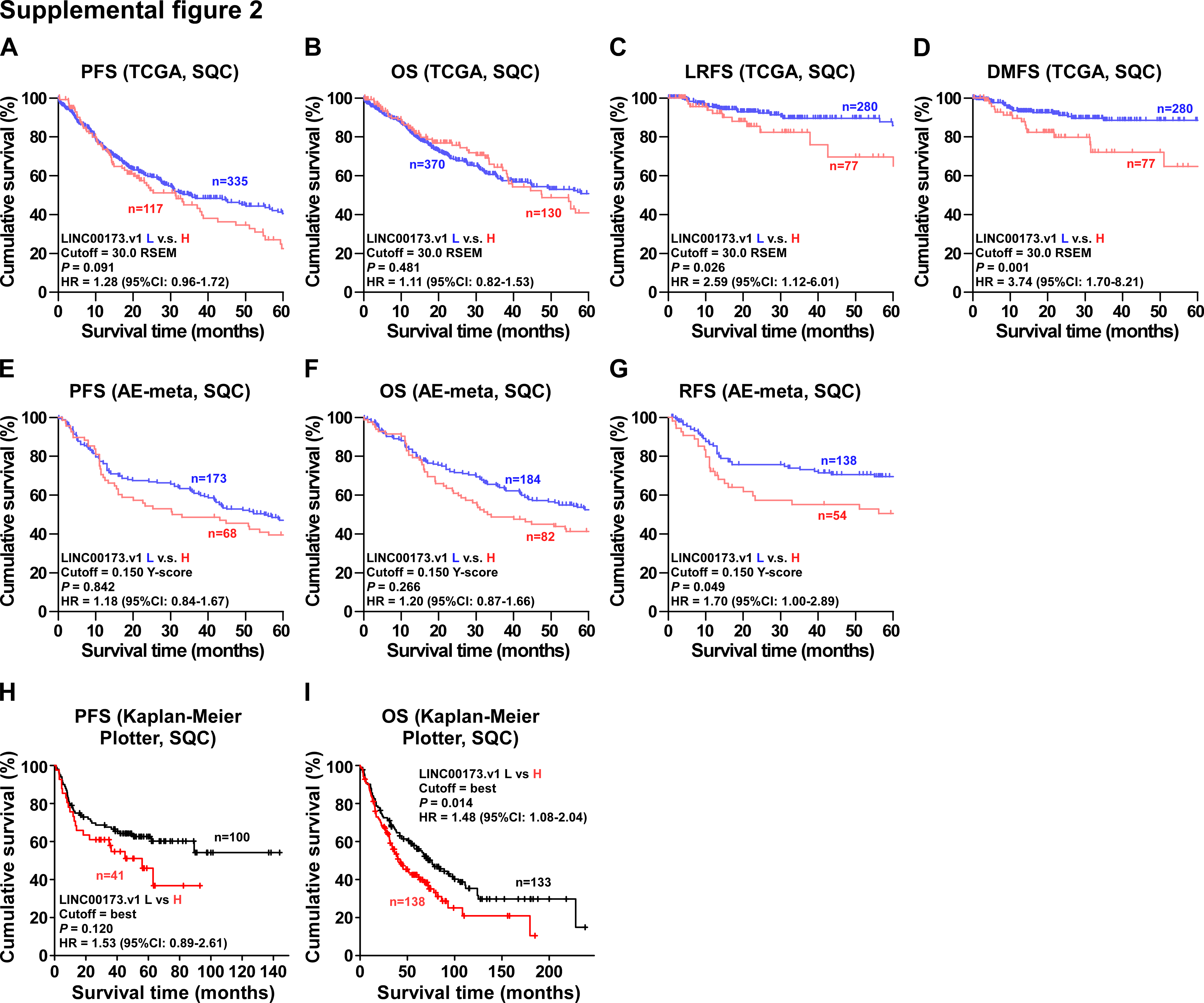

Supplement: Supplementary file 2 — Additional file 2: Supplement Figure 2. (a to d) Kaplan–Meier analysis of of progression-free survivals (PFS), overall survival (OS), local relapse-free survival (LRFS) and distant metastasis-free survival (DMFS) in SQC patients with low LINC00173.v1 expression versus high LINC00173.v1 expression from TCGA. P value was determined by Log-rank test. HR indicates hazard ratio; 95%CI indicates 95% confidence interval. (e to g) Kaplan–Meier analysis of PFS, OS and relapse-free survival (RFS) in SQC patients with low LINC00173.v1 expression versus high LINC00173.v1 expression from AE-meta. P value was determined by Log-rank test. HR indicates hazard ratio; 95%CI indicates 95% confidence interval. (h and i) Kaplan-Meier Plotter of PFS and OS in SQC patients with low LINC00173.v1 expression versus high LINC00173.v1 expression. P value was determined by Log-rank test. HR indicates hazard ratio; 95%CI indicates 95% confidence interval. [file 12943_2020_1217_MOESM2_ESM.tif]

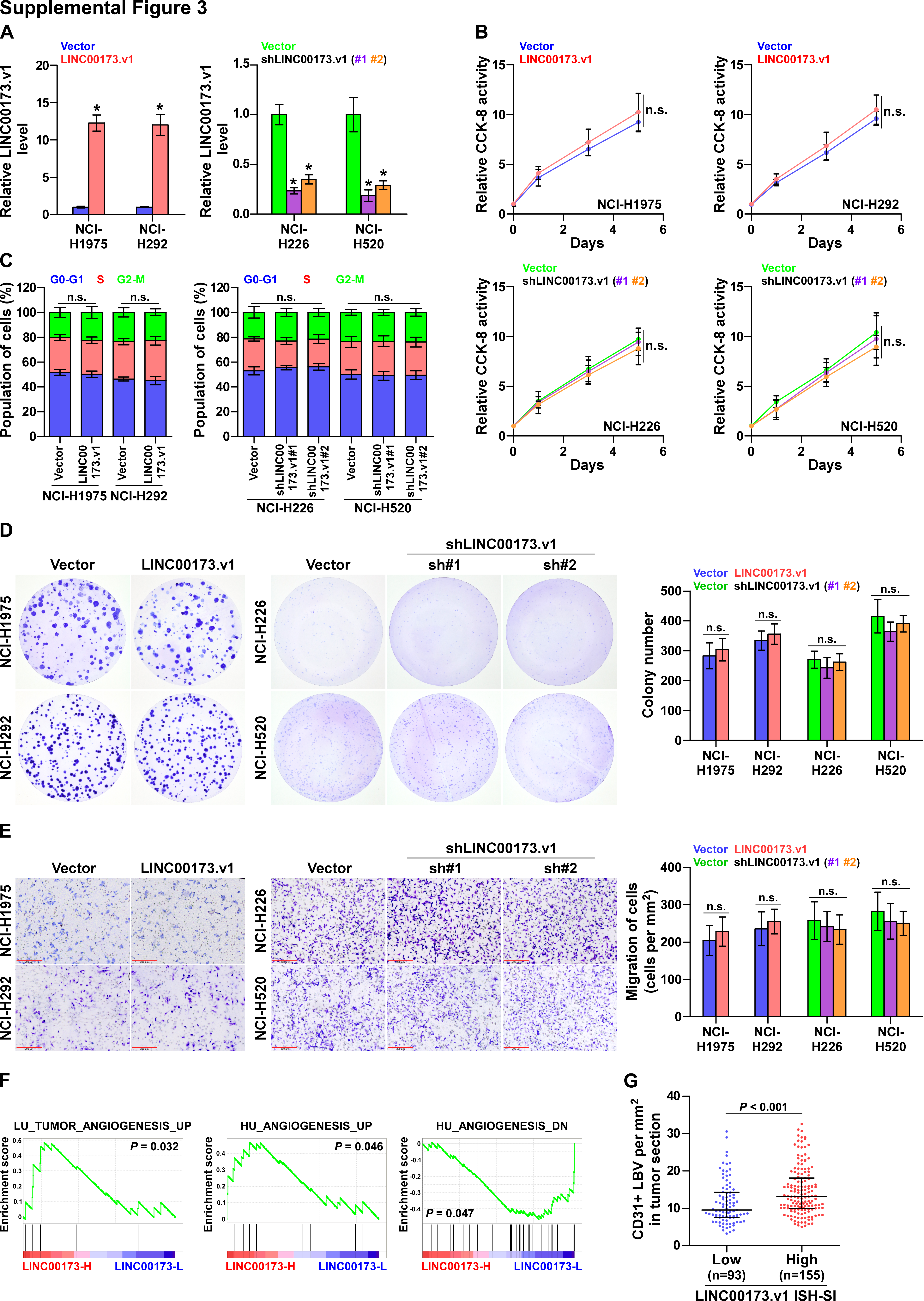

Supplement: Supplementary file 3 — Additional file 3: Supplement Figure 3. LINC00173.v1 does not interfere proliferation and migration ability of lung cancer cells in vitro. (a) Real-time PCR analysis of LINC00173.v1 expression in exogenously overexpressed LINC00173.v1 in ADC cell line NCI-H1975 and NSCLC cell line NCI-H292, and LINC00173.v1-stably downexpressing NCI-H226 and NCI-H520 SQC cells lines. Each bar represents the mean values ± SD of three independent experiments. *P < 0.05 by unpaired t test or one-way ANOVA test. (b) The effect of LINC00173.v1 on proliferation of lung cancer cells was assessed by CCK-8 assay. Each bar represents the mean values ± SD of three independent experiments. P value was determined by unpaired t test or one-way ANOVA test. n.s. indicates no significance. (c) The effects of LINC00173.v1 on the cell cycle progression of lung cancer cells. Each bar represents the mean values ± SD of three independent experiments. P value was determined by unpaired t test or one-way ANOVA test. n.s. indicates no significance. (d) The influence of LINC00173.v1 on proliferation of lung cancer cells was assessed by colony formation assay. Each bar represents the mean values ± SD of three independent experiments. P value was determined by unpaired t test or one-way ANOVA test. n.s. indicates no significance. (e) The influence of LINC00173.v1 on migration ability of lung cancer cells was assessed by Transwell assay. Each bar represents the mean values ± SD of three independent experiments. P value was determined by unpaired t test or one-way ANOVA test. n.s. indicates no significance. Scale bars, 200 μm. (f) Gene Set Enrichment Analysis (GSEA) of correlation with proliferation and migration of vascular endothelial cells-associated genes signatures based on LINC00173.v1 expression data from the TCGA. (g) CD31+ lymphatic or blood vessel (LBV) density in low and high expression SQC tissues. Each bar represents the median values ± quartile values. P value was determined by unpaired t test. [file 12943_2020_1217_MOESM3_ESM.tif]

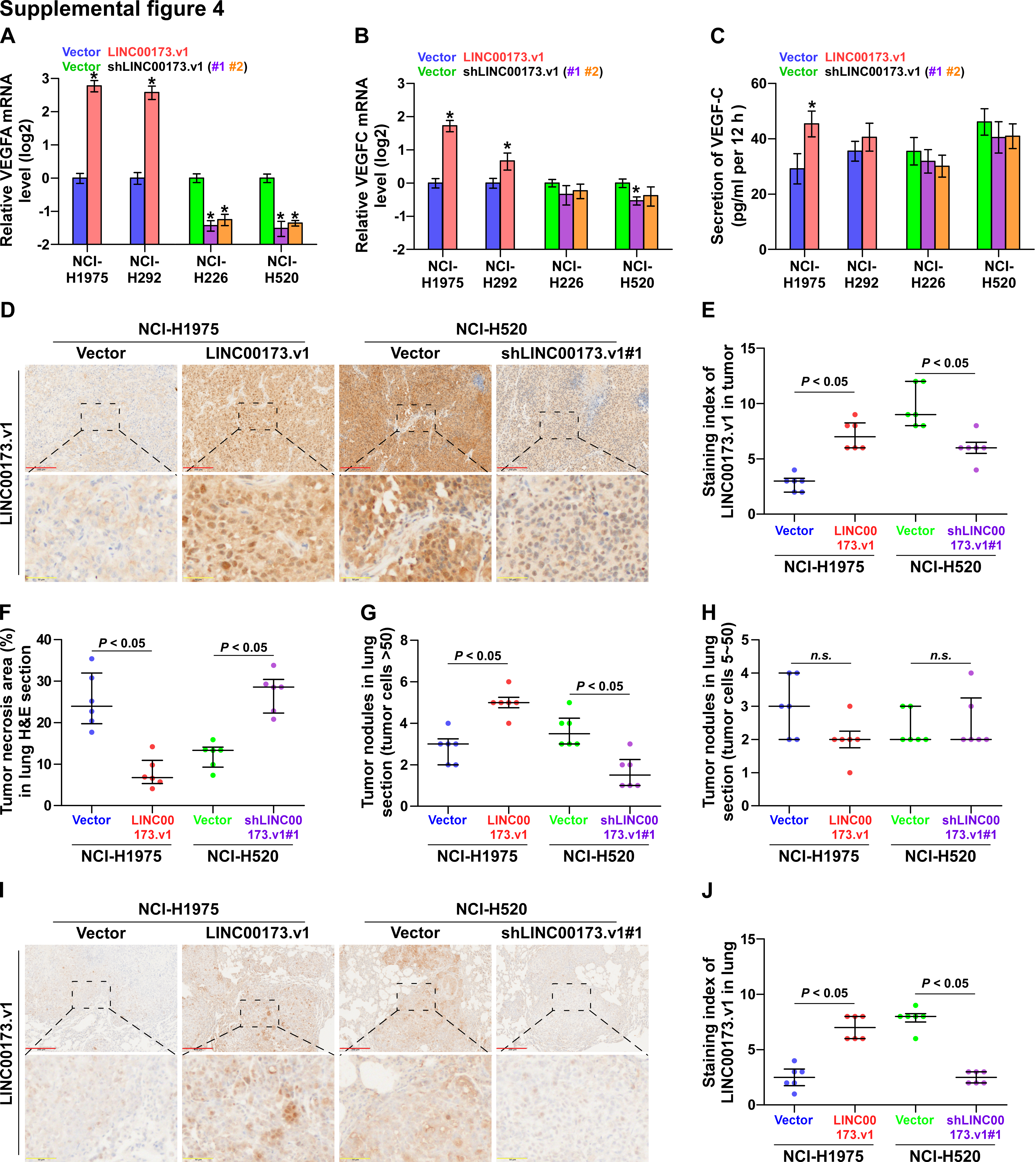

Supplement: Supplementary file 4 — Additional file 4: Supplement Figure 4. (a) Effect of LINC00173.v1 on mRNA levels of VEGFA in lung cancer cells by RT-PCR analysis. Each bar represents the mean values ± SD of three independent experiments. *P < 0.05 by unpaired t test or one-way ANOVA test. (b) Effect of LINC00173.v1 on mRNA levels of VEGFC in lung cancer cells by RT-PCR analysis. Each bar represents the mean values ± SD of three independent experiments. *P < 0.05 by unpaired t test or one-way ANOVA test. (c) Effect of LINC00173.v1 on secretion level of VEGF-C in lung cancer cells by enzyme linked immunosorbent assay (ELISA). Each bar represents the mean values ± SD of three independent experiments. *P < 0.05 by unpaired t test or one-way ANOVA test. (d and e) Representative images and staining index of LINC00173.v1 in tumor tissues from the indicated mice groups (n = 6) after 5 weeks of cell injection. Each bar represents the median values ± quartile values. P value was determined by unpaired t test. Scale bars of 100× magnification, 200 μm and 400× magnification, 50 μm. (f) Necrotic area in tumor tissues from the indicated mice groups after 5 weeks of cell injection. Each bar represents the median values ± quartile values. P value was determined by unpaired t test. (g and h) Tumor nodules of tumor cells > 50 (d) and < 50 (e) in lung sections from the indicated mice groups after 5 weeks of cell injection. Each bar represents the median values ± quartile values. P value was determined by unpaired t test. n.s. indicates no significance. (i and j) Representative sections and staining index of LINC00173.v1 in metastatic lung tumor tissues from the indicated mice groups (n = 6). Each bar represents the median values ± quartile values. P value was determined by unpaired t test. Scale bars of 100× magnification, 200 μm and 400× magnification, 50 μm. [file 12943_2020_1217_MOESM4_ESM.tif]

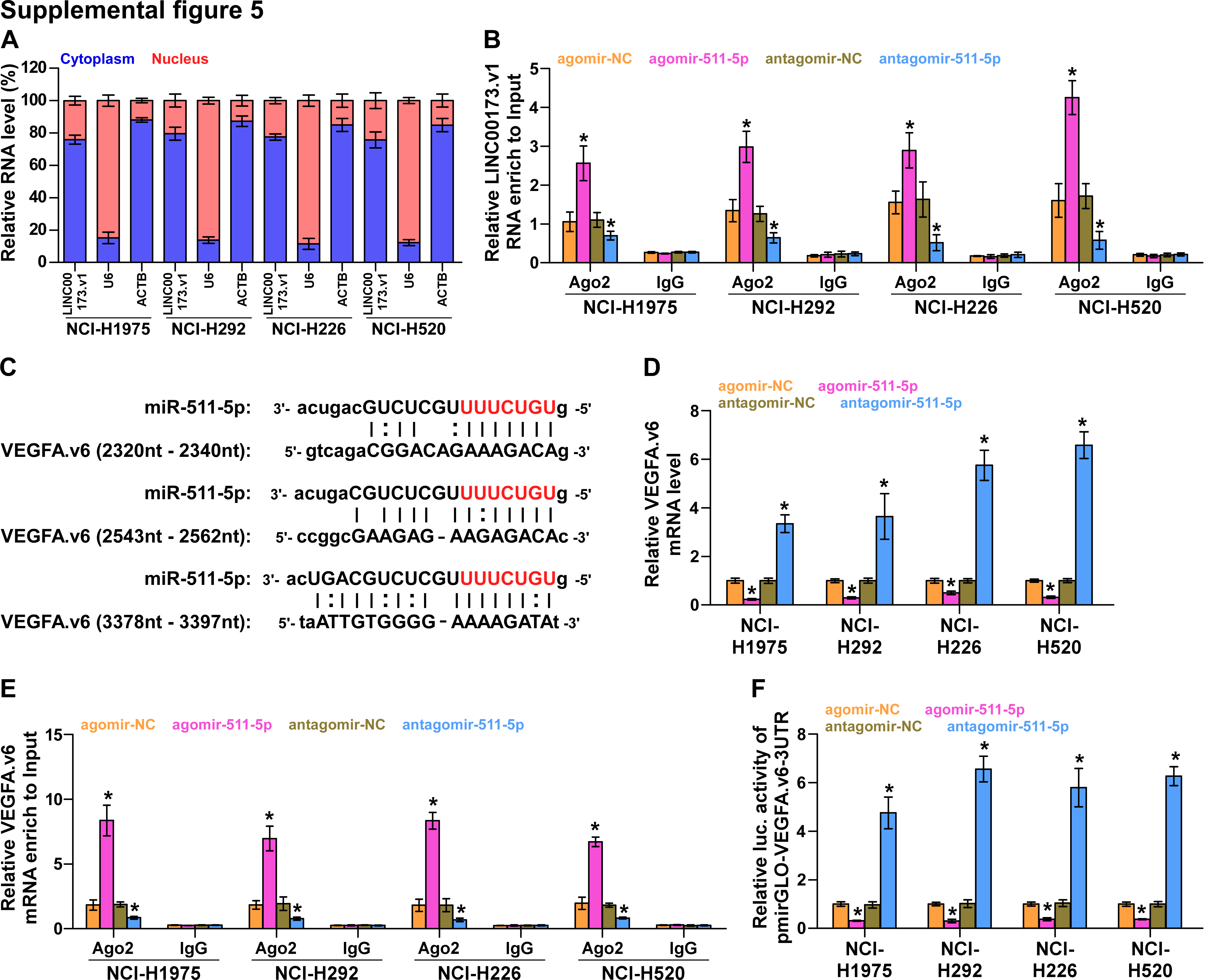

Supplement: Supplementary file 5 — Additional file 5: Supplement Figure 5. (a) Nuclear–cytoplasmic fractionation assays revealed that LINC00173.v1 was abundant in cytoplasm of lung cancer cells. U6 and actin were used as positive controls in nucleus and cytoplasm, respectively. Each bar represents the mean values ± SD of three independent experiments. (b) RNA immunoprecipitation (RIP) assay of the enrichment of LINC00173.v1 on miR-511-5p. IgG was used as negative control. Each bar represents the mean values ± SD of three independent experiments. *P < 0.05 by one-way ANOVA test. (c) Predicted miR-511-5p target sequence in 3’UTRs of VEGFA.v6. (d) RT-qPCR analysis of the effect of miR-511-5p on VEGFA.v6 in the indicated cells. Transcript levels were normalized by GAPDH expression. Each bar represents the mean values ± SD of three independent experiments. *P < 0.05 by one-way ANOVA test. (e) RNA immunoprecipitation (RIP) assay of the enrichment of miR-511-5p on VEGFA.v6. IgG was used as negative control. Each bar represents the mean values ± SD of three independent experiments. *P < 0.05 by ANOVA for repeated measures. (f) The influence of miR-511-5p on luciferase reporter activity of VEGFA.v6. Each bar represents the mean values ± SD of three independent experiments. *P < 0.05 by one-way ANOVA test. [file 12943_2020_1217_MOESM5_ESM.tif]

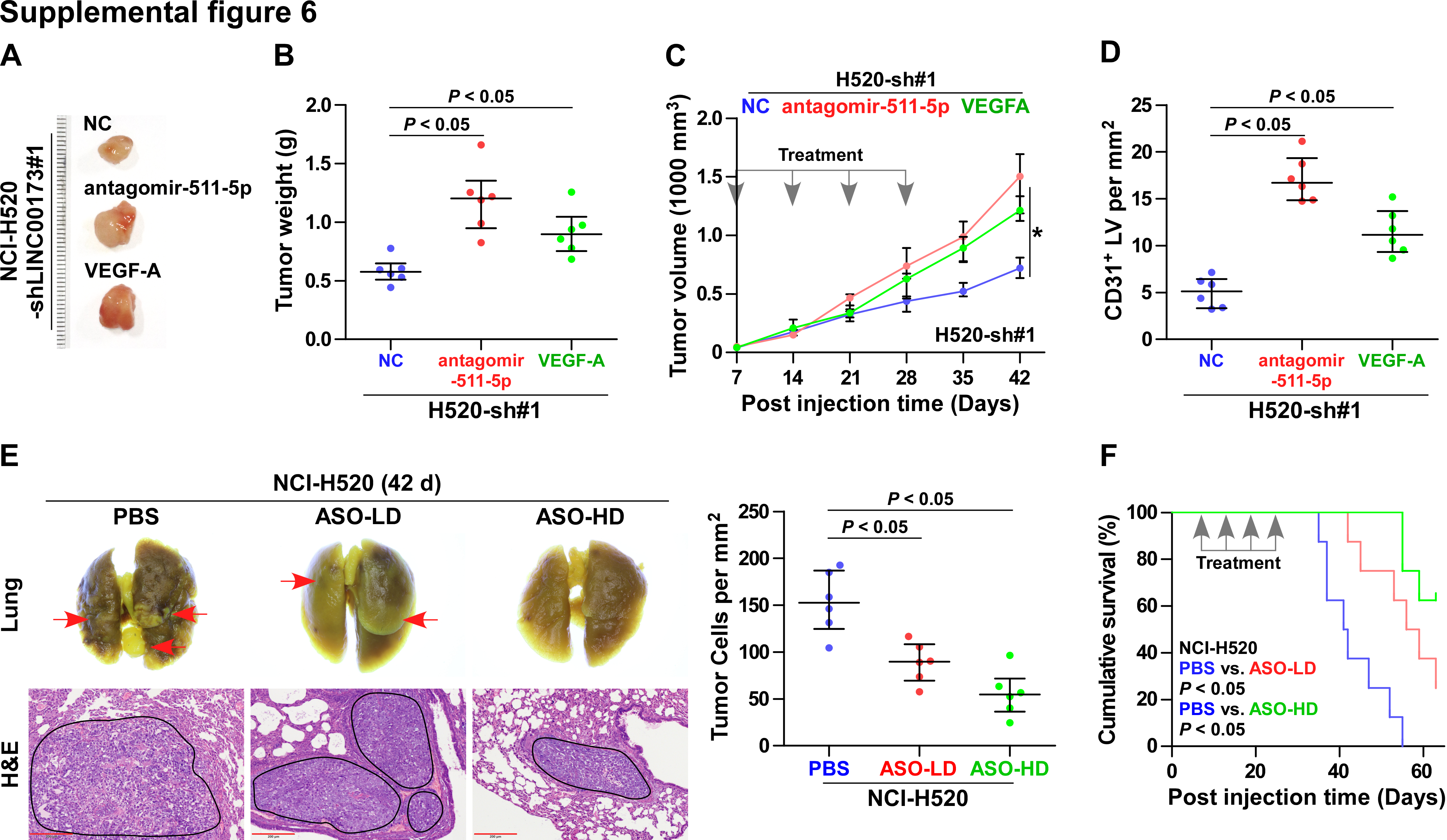

Supplement: Supplementary file 6 — Additional file 6: Supplement Figure 6. (a) Images of the representative excised tumors from the indicated mice (n = 6) at 42 days after injection. (b) Average weight of excised tumors from the indicated mice (n = 6). Each bar represents the median values ± quartile values. *P < 0.05 by one-way ANOVA. (c) Tumor volumes were measured every 7 days. Each bar represents the median values ± quartile values. P value was determined by ANOVA test in final measurement. (d) Density of CD31+ lymphatic or blood vessel (LBV) in tumor tissues from the indicated mice groups. Each bar represents the median values ± quartile values. P value was determined by ANOVA test. (e) Metastatic lung tumor nests in the mice groups injected with PBS, low- and high-dose LINC00173.v1 ASO Lung metastatic tumor tissues in mice were confirmed by H&E staining (Left panel). Tumor cell number per mm2 in lung H&E section from the indicated mice groups after 6 weeks of tail veins injection (Right panel). Each bar represents the median values ± quartile values. P value was determined by ANOVA test. Scale bars, 200. (f) Kaplan–Meier analysis of the effect of PBS, low- and high-dose LINC00173.v1 ASO on cumulative survival in the indicated mice groups (n = 8). P value was determined by Log-rank test. [file 12943_2020_1217_MOESM6_ESM.tif]

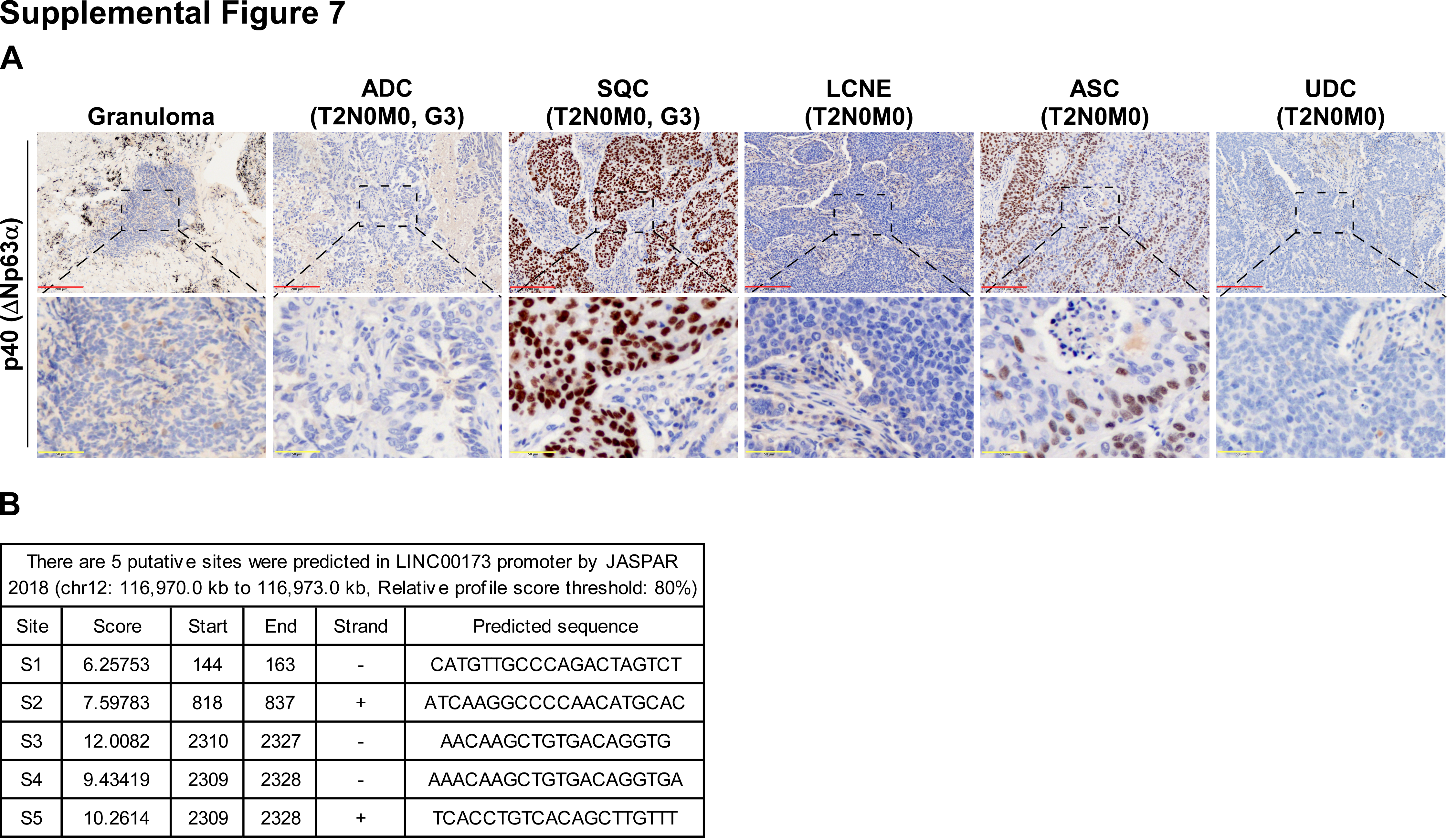

Supplement: Supplementary file 7 — Additional file 7: Supplement Figure 7. Squamous cell carcinoma-specific factor ΔNp63α contributes to LINC00173.v1 overexpression in SQC (a) Representative sections of ΔNp63α in 43 benign lung tissues (eg. granuloma), 248 SQC tissues, 122 ADC tissues and 26 other subtypes of lung cancer including LCNE, ASC, and UDC, using immunohistochemical staining (IHC). Scale bars of 100× magnification, 200 μm and 400× magnification, 50 μm. (b) The putative binding sites of ΔNp63α in LINC00173 promoters by JASPAR. [file 12943_2020_1217_MOESM7_ESM.tif]
