# Supplementary material for: LINC00173.v1 promotes angiogenesis and progression of lung squamous cell carcinoma by sponging miR-511-5p to regulate VEGFA expression
Source: Mol Cancer. 2020 May 30;19:98. doi: 10.1186/s12943-020-01217-2 (PMC7260858; doi:10.1186/s12943-020-01217-2)
Supplement: Supplementary file 8 — Additional file 8: Supplemental Table 1. The basic information of 20 NSCLC patients for LINC00173.v1 RT-qPCR analysis. Supplemental Table 2. The basic information of 43 patients with benign lung disease for LINC00173.v1 in situ hybridization analysis. Supplemental Table 3. The basic information of 396 patients with NSCLC for LINC00173.v1 in situ hybridization analysis. Supplemental Table 4. A list of primers used in the reactions for clone PCR. Supplemental Table 5. A list of primers used in the reactions for RT-qPCR. Supplemental Table 6. A list of primers used in the reactions for CHIP assay. Supplemental Table 7. The relationship between LINC00173.v1 expression level and clinical pathological characteristics in 396 patients with NSCLC. Supplemental Table 8. The relationship between LINC00173.v1 expression level and clinical pathological characteristics in 248 patients with lung SQC. [file 12943_2020_1217_MOESM8_ESM.docx]

### Supplemental Information

#### Supplemental Material and methods

##### RNA extraction, reverse transcription, and real-time quantitative PCR

Total RNA from tissues or cells was extracted using RNA Isolater (Vazyme, China) according to the manufacturer’s instructions. Messenger RNA (mRNA) and miRNA was reverse transcribed of total mRNA using the HiScript III 1st Strand cDNA Synthesis Kit (Vazyme, China) according to the manufacturer’s protocol. Complementary DNA (cDNA) was amplified and quantified on ABI 7500 Fast system (Thermo-Fisher, USA) using ChamQ Universal SYBR qPCR Master Mix (Vazyme, China). The mRNA primers were provided in **Supplemental table 5**, and were synthesized and purified by Biosune Biotechnology Co., Ltd (China), and primers for U6 and miRs were synthesized and purified by RiboBio (China). Quantitative PCR was performed according to the manufacturer’s protocol and was performed as previously described ^[1]^. Glyceraldehyde-3-phosphate dehydrogenase (GAPDH) was used as endogenous controls for mRNA, and U6 as endogenous controls for miRNA quantify. Relative fold expressions were calculated with the comparative threshold cycle (2^-ΔΔCt) method.

##### Nuclear-cytoplasmic fractionation assay

Nucleus and cytoplasm segmentation PARISTM Kit (Ambion, Austin, TX, USA) were used to analyze the nuclear or cytoplasmic fractionation of LINC00173.v1 expression in lung cancer cells as previously described ^[2]^.

##### Dual luciferase report experiments

Cells (5 ×10^5) were plated in 60 mm cell culture dishes, proliferating to 60-80% confluence after 24 h of culture, and the reporter constructs were transfected into cells using Lipofectamine 3000. After 36 h incubation, the transfection medium was replaced, and the luciferase assay was performed as previously described ^[3]^. Briefly, cells were harvested and washed with PBS, and lysed with lysis buffer. The cell lysates were analyzed immediately using Synergy 2 microplate system (BioTek, USA). Luciferase and Renilla luciferase were measured using a Renilla-Lumi Luciferase Reporter Gene Assay Kit (Beyotime, China) according to the manufacturer’s instructions. The luciferase activity of each lysate was normalized to Renilla luciferase activity. The relative transcriptional activity was converted into fold induction above the control group value.

##### Enzyme-linked immunosorbent assay (ELISA)

ELISA was performed as previously described ^[4]^. Briefly, cells (5 ×10^5) were seeded into 6-well plates and incubated for 24h at 37°C in 5% CO_2_ atmosphere. Then the conditional media (without FBS) was collected and used in order to determine the secretion of VEGFA, using the VEGF-A ELISA kit (Raybiotech, China) and VEGF-C ELISA kit (Raybiotech, China) in accordance with the manufacturer's protocol. Absorbance was measured on a Synergy 2 microplate system (BioTek, USA) at 450 nm.

##### RNA immunoprecipitation (RIP)

RIP was performed as previously described ^[1]^. Briefly, cells were co-transfected with HA-Ago2 (#10822; Addgene, USA), followed by HA-Ago2 immunoprecipitation using HA-antibody (Cell Signaling Technology, USA). Real-time PCR analysis of the immunoprecipitation material was used to test the association of the miR-511-5p and LINC00173.v1, VEGFA.v6 with the RNA-induced silencing complex (RISC complex).

##### Chromatin immunoprecipitation (ChIP)

ChIP was performed as previously described ^[5]^.Cells (6 ×10^6) in a 100-mm culture dish were treated with 1% formaldehyde to cross-link proteins to DNA. The cell lysates were sonicated to shear DNA to sizes of 300 to 1,000 bp. Equal aliquots of chromatin supernatants were incubated with 1 mg of anti-FLAG antibody (Cell Signaling Technology, USA) or an anti-IgG antibody (Cell Signaling Technology, USA) overnight at 4°C with rotation. After reverse cross-link of protein/DNA complexes to free DNA, real-time PCR was carried out. The primers used to detect DNA fragment in ChIP were provided in **Supplemental table 6**.

#### Reference

[1] Zhang X, Ren D, Wu X*, et al.* miR-1266 Contributes to Pancreatic Cancer Progression and Chemoresistance by the STAT3 and NF-kappaB Signaling Pathways [J]. Mol Ther Nucleic Acids, 2018, 11(1): 142-158.

[2] Lang C, Dai Y, Wu Z*, et al.* SMAD3/SP1 complex-mediated constitutive active loop between lncRNA PCAT7 and TGF-beta signaling promotes prostate cancer bone metastasis [J]. Mol Oncol, 2020.

[3] Zhang X, Zhang L, Lin B*, et al.* Phospholipid Phosphatase 4 promotes proliferation and tumorigenesis, and activates Ca(2+)-permeable Cationic Channel in lung carcinoma cells [J]. Mol Cancer, 2017, 16(1): 147.

[4] Zhang X, Ren D, Guo L*, et al.* Thymosin beta 10 is a key regulator of tumorigenesis and metastasis and a novel serum marker in breast cancer [J]. Breast Cancer Res, 2017, 19(1): 15.

[5] Chen J, Liu A, Lin Z*, et al.* Downregulation of the Circadian Rhythm Regulator HLF Promotes Multiple-Organ Distant Metastases in Non-Small Cell Lung Cancer through PPAR/NF-kappaB Signaling [J]. Cancer Lett, 2020, 482(1): 56-71.

**Supplemental table 1.** The basic information of 20 NSCLC patients for LINC00173.v1 RT-qPCR analysis.

|  | | Cases (n) | Percentage (%) |
| --- | --- | --- | --- |
| Histologic | ADC | 10 | 50 |
|  | SQC | 10 | 50 |
| Gender | Male | 12 | 60 |
|  | Female | 8 | 40 |
| Age | <60 | 11 | 55 |
|  | ≥60 | 9 | 45 |
| Grade | G1 | 0 | 0 |
|  | G2 | 14 | 70 |
|  | G3 | 6 | 30 |
| Stage | Stage I | 3 | 15 |
|  | Stage II | 12 | 60 |
|  | Stage III | 5 | 25 |
|  | Stage IV | 0 | 0 |

* ADC: Adenocarcinoma; SQC: Squamous carcinoma.

**Supplemental table 2.** The basic information of 43 patients with benign lung disease for LINC00173.v1 in situ hybridization analysis.

|  | | Cases (n) | Percentage (%) |
| --- | --- | --- | --- |
| Gender | Male | 33 | 76.7 |
|  | Female | 10 | 23.3 |
| Age | <60 | 34 | 79.1 |
|  | ≥60 | 9 | 20.9 |
| Type of diseases | Amyloidosis | 1 | 2.3 |
|  | Fungal infection | 2 | 4.7 |
|  | Hamartoma | 3 | 7.0 |
|  | Hyperplasia | 19 | 44.2 |
|  | Metaplasia | 1 | 2.3 |
|  | Pneumonia | 5 | 11.6 |
|  | Pulmonary bulla | 5 | 11.6 |
|  | Sclerosing hemangioma | 1 | 2.3 |
|  | Tuberculosis | 6 | 14.0 |

**Supplemental table 3.** The basic information of 396 patients with NSCLC for LINC00173.v1 in situ hybridization analysis.

|  | | Cases (n) | Percentage (%) |
| --- | --- | --- | --- |
| Histologic | ADC | 122 | 30.8 |
|  | SQC | 248 | 62.6 |
|  | Other | 26 | 6.6 |
| Gender | Female | 145 | 36.6 |
|  | Male | 251 | 63.4 |
| Age | <60 | 91 | 23.0 |
|  | ≥60 | 305 | 77.0 |
| Grade (excluded other type) | G1 | 38 | 10.3 |
|  | G2 | 182 | 49.2 |
|  | G3 | 150 | 40.5 |
| T classification | T1 | 106 | 26.8 |
|  | T2 | 201 | 50.8 |
|  | T3 | 66 | 16.7 |
|  | T4 | 22 | 5.6 |
|  | NA | 1 | 0.3 |
| N classification | N0 | 236 | 59.6 |
|  | N1 | 109 | 27.5 |
|  | N2 | 42 | 10.6 |
|  | N3 | 5 | 1.3 |
|  | NA | 4 | 1.0 |
| M classification | M0 | 372 | 93.9 |
|  | M1 | 24 | 6.1 |
| Stage | Stage I | 158 | 39.9 |
|  | Stage II | 132 | 33.3 |
|  | Stage III | 82 | 20.7 |
|  | Stage IV | 24 | 6.1 |

* ADC: Adenocarcinoma; SQC: Squamous carcinoma; NA: Not available.

** Other histologic cancers included: adenosquamous carcinoma (ASC), large cell neuroendocrine carcinoma (LCNE), undifferentiated carcinoma (UDC), epidermoid carcinoma (EPC), sarcomatoid carcinoma (SARC), and pleomorphic carcinoma (PMC).

**Supplemental table 4.** A list of primers used in the reactions for clone PCR.

| Gene | Sequence (5` – 3`) | |
| --- | --- | --- |
| VEGFA.v6-3`UTR -up | | AGGGAAGAGGAGGAGATGAGAG |
| VEGFA.v6-3`UTR -dn | | TGCACTAGAGACAAAGACGTGATG |
| sh LINC00173.v1#-up | | CCGGCACCTTGCTCCGCTGTTCTTTCTCGAGAAAGAACAGCGGAGCAAGGTGTTTTTG |
| sh LINC00173.v1-1#-dn | | AATTCAAAAACACCTTGCTCCGCTGTTCTTTCTCGAGAAAGAACAGCGGAGCAAGGTG |
| sh LINC00173.v1-2#-up | | CCGGTGGGATGTCAGAGGTGTTGATCTCGAGATCAACACCTCTGACATCCCATTTTTG |
| sh LINC00173.v1-2#-dn | | AATTCAAAAATGGGATGTCAGAGGTGTTGATCTCGAGATCAACACCTCTGACATCCCA |

**Supplemental table 5.** A list of primers used in the reactions for RT-qPCR.

| Gene name | Sequence (5` – 3`) | |
| --- | --- | --- |
| LINC00173.v1 | forward | TTCTGGGTCCGAGGCTCC |
|  | reverse | AGCTTTGCTCTTGCACTGAGATG |
| LINC00173.v2 | forward | TGGAAATTGGCAGGGTGAGAG |
|  | reverse | GGGAGGCAGAGGTGATCAAG |
| VEGFA.v6 | forward | TCTTCCAGGAGTACCCTGATGAG |
|  | reverse | GCTGGCCTTGGTGAGGTTTG |
| VEGFB | forward | CAAGTCCGGATGCAGATCCTC |
|  | reverse | TCTGGCTTCACAGCACTGTC |
| VEGFC | forward | GGCTGGCAACATAACAGAGAA |
|  | reverse | CCCCACATCTATACACACCTCC |
| VEGFD | forward | TCCCATCGGTCCACTAGGTTTG |
|  | reverse | ACCACATCGGAACACGTTCAC |
| EPO | forward | GGAGGCCGAGAATATCACGAC |
|  | reverse | CCCTGCCAGACTTCTACGG |
| PDGFA | forward | CGGATACCTCGCCCATGTTC |
|  | reverse | CTCTCAGGCTGGTGTCCAAAG |
| GAPDH | forward | GCACCGTCAAGGCTGAGAAC |
|  | reverse | TGGTGAAGACGCCAGTGGA |

**Supplemental table 6.** A list of primers used in the reactions for CHIP assay.

| Gene name | Sequence (5` – 3`) | |
| --- | --- | --- |
| LINC00173.v1-P1 | forward | AGCTCACACCTGTAATCCCC |
|  | reverse | CCTCCTGCCTCAGCTTCTTG |
| LINC00173.v1-P2 | forward | CGTTTCTTTGTGCCTGTGTTCC |
|  | reverse | CAGCAACGTGATCTGTGTTGTC |
| LINC00173.v1-P3 | forward | CGCCTTCTGTCTGTCTAGGTG |
|  | reverse | AGCCTGTCATATGGTGCTCTC |
| LINC00173.v1-P4 | forward | GTCTGTCGGGAATACTCGGTCT |
|  | reverse | GCCAGCATTGGGGATGTGTAATTG |

**Supplemental table 7.** The relationship between LINC00173.v1 expression level and clinical pathological characteristics in 396 patients with NSCLC.

| Parameters | Number of cases | LINC00173.v1 ISH expression | | *P* values |
| --- | --- | --- | --- | --- |
|  |  | Low (n=223) | High (n=173) |  |
| Histologic |  |  |  |  |
| ADC | 122 | 109 | 13 | <0.001* |
| SQC | 248 | 93 | 155 |  |
| Gender |  |  |  |  |
| Female | 145 | 90 | 55 | 0.093 |
| Male | 251 | 133 | 118 |  |
| Age |  |  |  |  |
| <60 | 91 | 56 | 35 | 0.279 |
| ≥60 | 305 | 167 | 138 |  |
| Grade |  |  |  |  |
| G1-G2 | 220 | 121 | 99 | 0.915 |
| G3 | 150 | 81 | 69 |  |
| T classification |  |  |  |  |
| T1-2 | 307 | 173 | 134 | 1.000 |
| T3-4 | 88 | 49 | 39 |  |
| N classification |  |  |  |  |
| N0 | 236 | 130 | 106 | 0.678 |
| N1-3 | 155 | 89 | 66 |  |
| M classification |  |  |  |  |
| M0 | 372 | 209 | 163 | 1.000 |
| M1 | 24 | 14 | 10 |  |
| Stage |  |  |  |  |
| I-II | 290 | 156 | 134 | 0.109 |
| III-IV | 106 | 67 | 39 |  |

* IHC: Immunohistochemistry; ADC: Adenocarcinoma; SQC: Squamous carcinoma.

**Supplemental table 8.** The relationship between LINC00173.v1 expression level and clinical pathological characteristics in 248 patients with lung SQC.

| Parameters | Number of cases | LINC00173.v1 ISH expression | | *P* values |
| --- | --- | --- | --- | --- |
|  |  | Low (n=93) | High (n=155) |  |
| Gender |  |  |  |  |
| Female | 85 | 38 | 47 | 0.091 |
| Male | 163 | 55 | 108 |  |
| Age |  |  |  |  |
| <60 | 52 | 23 | 29 | 0.259 |
| ≥60 | 196 | 70 | 126 |  |
| Grade |  |  |  |  |
| G1-G2 | 144 | 56 | 88 | 0.595 |
| G3 | 104 | 37 | 67 |  |
| T classification |  |  |  |  |
| T1-2 | 193 | 74 | 119 | 0.608 |
| T3-4 | 55 | 19 | 36 |  |
| N classification |  |  |  |  |
| N0 | 153 | 57 | 96 | 0.952 |
| N1-3 | 93 | 35 | 58 |  |
| M classification |  |  |  |  |
| M0 | 234 | 89 | 145 | 0.478 |
| M1 | 14 | 4 | 10 |  |
| Stage |  |  |  |  |
| I-II | 191 | 72 | 119 | 0.907 |
| III-IV | 57 | 21 | 36 |  |

* IHC: Immunohistochemistry.
